# Experimental Evolution Generates Novel Oncolytic Vesicular Stomatitis Viruses with Improved Replication in Virus-Resistant Pancreatic Cancer Cells

**DOI:** 10.1128/JVI.01643-19

**Published:** 2020-01-17

**Authors:** Sara L. Seegers, Connor Frasier, Sarah Greene, Irina V. Nesmelova, Valery Z. Grdzelishvili

**Affiliations:** a Department of Biological Sciences, University of North Carolina at Charlotte, Charlotte, North Carolina, USA; b Department of Physics and Optical Science, University of North Carolina at Charlotte, Charlotte, North Carolina, USA; University of Kentucky College of Medicine

**Keywords:** vesicular stomatitis virus, oncolytic virus, pancreatic cancer, pancreatic ductal adenocarcinoma, p53, experimental evolution, VSV glycoprotein, attachment, LDLR, transgene stability

## Abstract

Vesicular stomatitis virus (VSV) based oncolytic viruses are promising agents against various cancers. We have shown that pancreatic ductal adenocarcinoma (PDAC) cell lines exhibit great diversity in susceptibility and permissibility to VSV. Here, using a directed evolution approach with our two previously described oncolytic VSV recombinants, VSV-p53wt and VSV-p53-CC, we generated novel oncolytic VSVs with an improved ability to replicate in virus-resistant PDAC cell lines. VSV-p53wt and VSV-p53-CC encode a VSV matrix protein (M) with a ΔM51 mutation (M-ΔM51) and one of two versions of a functional human tumor suppressor, p53, fused to a far-red fluorescent protein, eqFP650. Each virus was serially passaged 32 times (which accounts for more than 60 viral replication cycles) on either the SUIT-2 (moderately resistant to VSV) or MIA PaCa-2 (highly permissive to VSV) human PDAC cell lines. While no phenotypic changes were observed for MIA PaCa-2-passaged viruses, both SUIT-2-passaged VSV-p53wt and VSV-p53-CC showed improved replication in SUIT-2 and AsPC-1, another human PDAC cell line also moderately resistant to VSV, while remaining highly attenuated in nonmalignant cells. Surprisingly, two identical VSV glycoprotein (VSV-G) mutations, K174E and E238K, were identified in both SUIT-2-passaged viruses. Additional experiments indicated that the acquired G mutations improved VSV replication, at least in part due to improved virus attachment to SUIT-2 cells. Importantly, no mutations were found in the M-ΔM51 protein, and no deletions or mutations were found in the p53 or eqFP650 portions of virus-carried transgenes in any of the passaged viruses, demonstrating long-term genomic stability of complex VSV recombinants carrying large transgenes.

**IMPORTANCE** Vesicular stomatitis virus (VSV)-based oncolytic viruses are promising agents against pancreatic ductal adenocarcinoma (PDAC). However, some PDAC cell lines are resistant to VSV. Here, using a directed viral evolution approach, we generated novel oncolytic VSVs with an improved ability to replicate in virus-resistant PDAC cell lines, while remaining highly attenuated in nonmalignant cells. Two independently evolved VSVs obtained 2 identical VSV glycoprotein mutations, K174E and E238K. Additional experiments indicated that these acquired G mutations improved VSV replication, at least in part due to improved virus attachment to SUIT-2 cells. Importantly, no deletions or mutations were found in the virus-carried transgenes in any of the passaged viruses. Our findings demonstrate long-term genomic stability of complex VSV recombinants carrying large transgenes and support further clinical development of oncolytic VSV recombinants as safe therapeutics for cancer.

## Abstract (precis)

Vesicular stomatitis virus (VSV)-based oncolytic viruses are promising agents against pancreatic ductal adenocarcinoma (PDAC). However, some PDAC cell lines are resistant to VSV. Here, using a directed viral evolution approach, we generated novel oncolytic VSVs with an improved ability to replicate in virus-resistant PDAC cell lines, while remaining highly attenuated in nonmalignant cells. Two independently evolved VSVs obtained 2 identical VSV glycoprotein mutations, K174E and E238K. Additional experiments indicated that these acquired G mutations improved VSV replication, at least in part due to improved virus attachment to SUIT-2 cells. Importantly, no deletions or mutations were found in the virus-carried transgenes in any of the passaged viruses. Our findings demonstrate long-term genomic stability of complex VSV recombinants carrying large transgenes and support further clinical development of oncolytic VSV recombinants as safe therapeutics for cancer.

## INTRODUCTION

Vesicular stomatitis virus (VSV) is a prototypic nonsegmented negative-strand (NNS) RNA virus (order *Mononegavirales*, family *Rhabdoviridae*, genus *Vesiculovirus*). The 11-kb genome of VSV encodes five proteins: nucleocapsid protein (N), phosphoprotein (P), matrix protein (M), glycoprotein (G), and large polymerase (L) (1). VSV is able to infect and replicate in a wide range of cell types (2) due to the use of ubiquitously expressed cell surface molecules. The low-density lipoprotein receptor (LDLR) and other members of the LDLR family have been shown to serve as VSV receptors (3 – 6), and additional studies showed that other cell surface molecules, such as phosphatidylserine (7 – 9), sialoglycolipids (10), and heparan sulfate (11) could also play a role in VSV attachment to host cells.

VSV is a promising oncolytic virus (OV) due to its inherent ability to preferentially replicate in cancer cells and because of a lack of preexisting immunity against VSV in the human population (12 – 14). The oncoselectivity of most OVs, including VSV, is mainly based on defective or reduced type I interferon (IFN) responses in cancer cells, compared to nonmalignant (“normal”) cells (15 – 23). These responses are generally unfavorable for tumor development as they are antiproliferative, anti-angiogenic, and proapoptotic (24). Although wild-type (WT) VSV is sensitive to type I IFN-mediated antiviral responses in most normal tissues, WT VSV-M sufficiently inhibits type I IFN responses to allow viral replication in the central nervous system (CNS) (25, 26). However, due to a well-established reverse genetics system available for VSV, a large number of safe oncoselective VSV-based oncolytic viruses have been generated and tested in numerous studies (12, 14). Some of the most widely used oncolytic VSVs are recombinants carrying a deletion (M-ΔM51) or substitution (M51R) of methionine at amino acid (aa) residue 51 in VSV-M. These mutations attenuate VSV replication in normal cells by preventing WT VSV-M protein from inhibition the nuclear exit of host mRNAs, including transcripts for virus-induced antiviral genes (27 – 29). As a result, unlike WT VSV, VSV-M51 mutants have dramatically attenuated neurotoxicity but retain robust oncolytic abilities (16, 30 – 35).

Another common approach to generate safe oncolytic VSVs is to introduce a transgene improving oncoselectivity or/and induction of adaptive anti-tumor immune responses (12 – 14). For example, previous studies showed that functional human tumor suppressor p53 variants can be successfully integrated into VSV genome (36, 37). Our laboratory generated two recombinants, VSV-p53wt and VSV-p53-CC, each expressing M-ΔM51 and a different version of a functional tumor suppressor p53 fused to a near-infrared fluorescent protein, eqFP650 (hereinafter referred to as RFP) (37). VSV-p53wt encodes a human WT p53, while VSV-p53-CC encodes a human p53 with its tetramerization domain substituted for with the coiled-coil (CC) domain of breakpoint cluster region (Bcr) protein (38). The resulting p53-CC protein evades the dominant-negative activities of endogenously expressed mutant p53 (38). Our previous study showed that these VSV-carried p53 transgenes not only enhanced VSV anticancer abilities through the introduction of functional p53 into cancer cells with defective tumor suppression activity, but also through the downregulation of antiviral signaling in cancer cells (37).

As a result of the numerous preclinical studies demonstrating the effectiveness of different VSV recombinants as OVs (12, 14, 39), VSV-hIFNbeta-NIS, encoding the human cytokine interferon beta (hIFNbeta) and the human thyroidal sodium-iodine symporter (NIS), is currently being tested in the United States in several phase I clinical trials against various malignancies. (For details see ClinicalTrials.gov for trials NCT03647163, NCT02923466, NCT03120624, NCT03865212, and NCT03017820.) Despite these advances, many challenges exist regarding the use of VSV as an oncolytic virus in the clinic. For example, not all tumors are susceptible and/or permissive to VSV (12, 14). Our previous studies showed that pancreatic ductal adenocarcinoma (PDAC) cell lines show great diversity in susceptibility and permissibility to VSV-based OVs, such as VSV-ΔM51. We previously identified several mechanisms behind resistance of PDACs to VSV-based therapy, such as abnormal or residual type I IFN antiviral activities (40 – 43), inefficient attachment of VSV to some PDACs (44), and resistance of VSV-infected PDAC cells to virus-mediated apoptosis (45).

Another potential problem is that VSV, as any other RNA virus, can mutate rapidly due to the lack of proofreading activities in virus-encoded RNA-dependent RNA polymerase (RdRp) (46). Such spontaneous mutations could revert attenuated VSV back to a WT phenotype. For example, in the case of VSV-ΔM51 recombinants, secondary mutations in VSV-M could hypothetically restore WT M functions and reduce VSV-ΔM51 oncoselectivity. Also, VSV has a small RNA genome, and the addition of any transgenes typically attenuates viral replication as the added genetic information hinders speed of viral genome replication and attenuates transcription of downstream viral genes (47). A spontaneous loss of a transgene, particularly if the transgene is the attenuating factor, is an undesirable possibility. Another hypothetical complication is single-site mutations in the beneficial transgene, which could completely negate or change its function, resulting in an ineffective or potentially pathogenic function. Thus, while WT p53 is a powerful tumor suppressor, when mutated, p53 can acquire devastating gain-of-function oncogenic activities, promoting cell survival, proliferation, invasion, migration, chemoresistance, tissue remodeling, and chronic inflammation (48, 49). Therefore, an important objective of this study was to experimentally examine the stability of VSV-carried transgenes and the retention of the safe oncoselective phenotype.

Here, we used an experimental evolution approach to obtain novel VSVs adapted to better replicate in virus-resistant PDAC cells and to examine the stability of VSV-carried transgenes after virus replication over an extended period of time. Directed evolution and bioselection for more potent oncolytic viruses has been explored in other studies using a variety of oncolytic viruses and cancer types (50 – 56). VSV has been a widely used model to study viral evolution for several decades (57) and has been experimentally evolved for various purposes, such as understanding how viruses evade innate immune responses (58) and the generation of novel VSV-G protein variants used to pseudotype retroviral and lentiviral vectors for gene delivery (59) and to produce novel variants of foreign proteins encoded in VSV genome (60). Moreover, several previous studies have successfully used a directed evolution approach to improve VSV’s oncolytic abilities (61 – 64). Using this approach in our current study, we generated 2 novel oncolytic VSVs with improved replication in VSV-resistant PDAC cell lines. Our findings also demonstrate long-term genomic stability of complex VSV recombinants carrying large transgenes.

## RESULTS

### Experimental evolution of two oncolytic VSV variants in two human PDAC cell lines.

**FIG 1:**
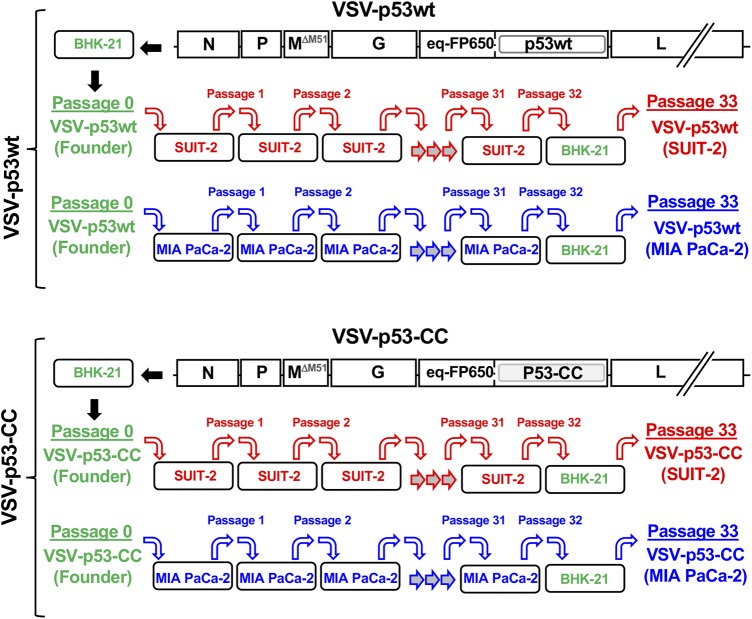
Scheme of viral passaging. Viruses VSV-p53wt (Founder) and VSV-p53-CC (Founder) (“Passage 0” indicates amplified in BHK-21 cells) were serially passaged independently 32 times on the PDAC cell line MIA PaCa-2 or SUIT-2. VSV-p53wt (Founder) and VSV-p53-CC (Founder) were added at an MOI of 0.1 to fresh cells for each passage. Cells were incubated with virus for 1 h, after which the virus was removed and fresh medium was added. Virus-containing supernatant was collected 24 h p.i., which was used for each subsequent viral passage (MOI of 0.1). Each virus had a final passage on BHK-21 cells, resulting in the following passage 33 viruses used throughout this study: VSV-p53wt (MIA PaCa-2), VSV-p53-CC (MIA PaCa-2), VSV-p53wt (SUIT-2), and VSV-p53-CC (SUIT-2).

Our experimental evolution experiments employed two different oncolytic VSV recombinants, VSV-p53wt and VSV-p53-CC, previously generated in our laboratory (37) (Fig. 1). Both viruses have a ΔM51 mutation in VSV-M and a transgene inserted between VSV-G and VSV-L and encoding the N terminus of p53 (p53wt or p53-CC) fused to the C terminus of RFP (65) (Fig. 1). Passaging two different VSV recombinants that have the same ΔM51 attenuation and RFP transgene but different p53 variants allowed us to ensure there was no viral cross-contamination over the course of the parallel viral passaging, as the differences between p53wt and p53-CC served as “molecular barcodes” for each recombinant virus while still offering a type of biological repeat since the viruses have shown very similar phenotypes (37).

We passaged 2 founder viruses VSV-p53wt (Founder) and VSV-p53-CC (Founder), which were produced by BHK-21 cells (a highly permissive baby hamster kidney cell line widely used for VSV amplification), in parallel on two different human PDAC cell lines, SUIT-2 and MIA PaCa-2 (Fig. 1). The cell lines SUIT-2 and MIA PaCa-2 were chosen because of their differential permissiveness to VSV and other differences. SUIT-2 cells are more resistant to VSV infection in part because of residual type I IFN responses, yet permissive enough to support sufficient viral replication to produce enough viral progeny for continued viral passaging, while MIA PaCa-2 cells are very permissive to VSV infection in part due to their inactive type I IFN signaling (37, 41, 66). Also, we showed that SUIT-2 cells showed lower levels of VSV attachment, compared to MIA PaCa-2 cells (44). Each virus has a large transgene (about 17% of the WT VSV genome) encoding a different version of p53 fused to RFP. Regarding p53, a previous study from our lab showed that VSV encoding p53 could stimulate VSV replication in cancer cells with active type I IFN signaling, such as SUIT-2 cells, but had no effect on VSV replication in MIA PaCa-2 cells that are defective in type I IFN signaling (37). The RFP reporter sequences are presumably dispensable for VSV replication in both cell lines, and previous studies suggest that the addition of a reporter transgene to VSV genome slightly attenuates viral replication (47). However, nucleotide substitutions or deletions in the RFP coding region could negatively affect p53 expression or function because the N terminus of p53 (p53wt or p53-CC) is fused to the C terminus of RFP in both viruses (65). In general, while we expected stronger selective pressures in SUIT-2 cells, both viruses could improve viral replication by losing at least some transgenic sequences due to random mutations as it would reduce the time it takes to replicate the viral genome and assemble virions.

For each passage, fresh uninfected cells were infected at a multiplicity of infection (MOI) of 0.1 PFU/ml (here and elsewhere in this study, the MOI was calculated based on titrating viruses on BHK-21 cells) by incubating fresh cells for 1 h with a previous virus passage, washing off any unbound virus, and incubating cells for an additional 23 h. A portion of the supernatant from the infected cells was collected 24 h postinfection (p.i.) to be used for the next passage, while the remaining supernatant was saved and stored at −80°C. After a final passage (passage 32) on a PDAC cell line, each virus was amplified on BHK-21 to generate the following four passage 33 viruses: VSV-p53wt (SUIT-2), VSV-p53-CC (SUIT-2), VSV-p53wt (MIA PaCa-2), and VSV-p53-CC (MIA PaCa-2). This final amplification on BHK-21 cells was done to generate stocks of virus particles comparable to the founder virus particles that were originally amplified on BHK-21 cells (Fig. 1).

### Viral genome sequence analysis of passaged viruses.

To examine if any mutations within coding (viral or transgenic) or noncoding regions of viral genomes took place over the course of the 33 passages, the genomes of each founder virus and passage 33 virus were fully sequenced using Sanger sequencing. Despite the advantages of next-generation sequencing techniques, Sanger sequencing allowed us to focus on major mutations that would become fixed or at least highly prevalent in viral populations by passage 33. For sequencing, supernatants containing viral particles were used to isolate viral genomic RNA that was reversed transcribed into cDNA using random hexamer primers. The generated cDNA was then PCR amplified to generate overlapping DNA products covering the entire viral genomes. (Primer sequences are not shown.)

**FIG 2:**
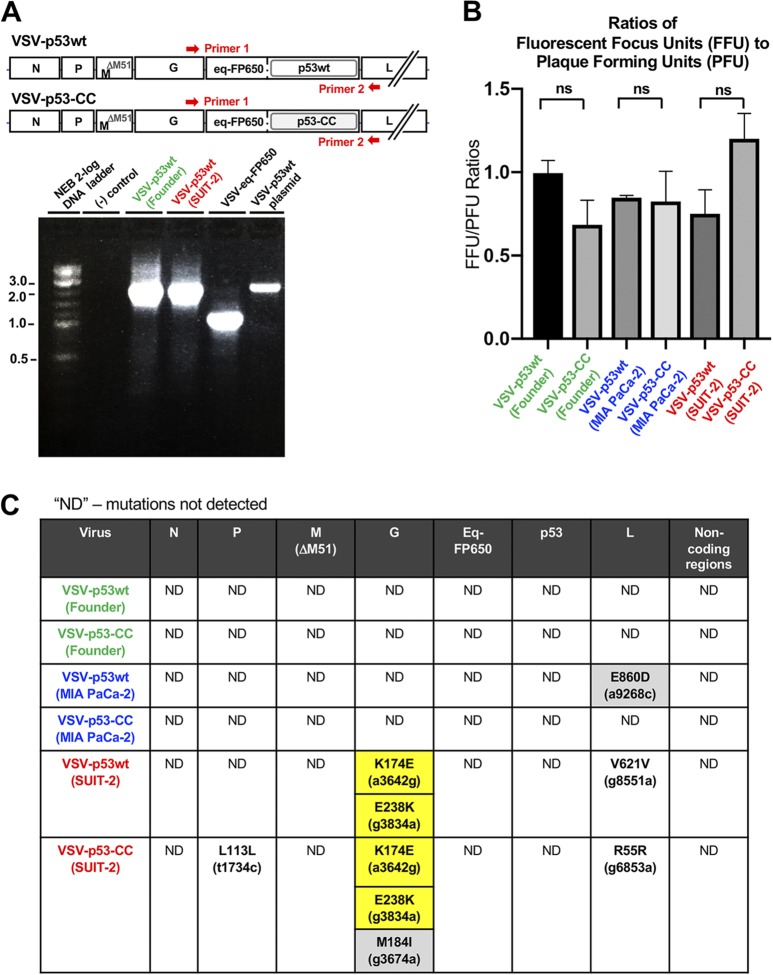
(A) Analysis of transgene-containing sequence between VSV-G and VSV-L to examine the stability of VSV recombinants carrying transgenes. Supernatants containing viral particles for the founder and passaged viruses were used to isolate viral genomic RNA that was reversed transcribed into cDNA using random hexamers. This cDNA was then amplified by PCR. Red arrows show an approximate position of annealing sites for VSV-specific primers located in the VSV-G and VSV-L regions. As controls, we used a plasmid containing a full-length copy of viral genome of VSV-p53wt and cDNA generated from VSV-eq-FP650 virus, which carries a shorter transgene (coding for RFP only, with no p53 sequences). (B) Comparison of the ratios of viral titers calculated by FFU divided by PFU indicates no loss of RFP transgene sequences from the viral genome after 33 passages. Results shown are representative of 2 independent repeats. Data shown represent the means and standard errors of the means (SEM). Results were analyzed to determine significance using one-way analysis of variance (ANOVA) with a Tukey posttest at a 95% confidence interval for comparison between each condition. All conditions tested were statistically insignificant, with no *P* value of <0.05. (C) The entire genomes for all founder and passage 33 viruses were sequenced using Sanger sequencing. Supernatants containing viral particles for the founder and passaged viruses were used to isolate viral genomic RNA, which was reversed transcribed into cDNA using random hexamers. This cDNA was then amplified by PCR. All identified mutations are listed in the table above. Silent mutations are denoted in black font whereas missense mutations are denoted in boldface black font and highlighted in gray if only present in one virus or highlighted in yellow if present in two viruses. The region of the viral genome where the mutations were identified is located at the top of the table.

To examine the stability of VSV-carried transgenes, we amplified, analyzed by size, and sequenced a portion of viral genome containing transgene sequences between VSV-G and VSV-L coding regions (Fig. 2A). As controls, we used a plasmid containing a full-length cDNA copy of the viral genome of VSV-p53wt (the VSV-p53wt plasmid in Fig. 2A) and cDNA generated from a VSV-eq-FP650 virus that carries a shorter transgene (RFP only, no p53 sequences) (VSV-eq-FP650 in Fig. 2A). If passaged viruses lost any significant portions of their transgenes, we expected to see shorter PCR fragments. In addition, all these PCR fragments were sequenced to detect any nucleotide changes in this region. We did not detect any deletions in the transgene regions in any of the passage 33 viruses (Fig. 2A) (data not shown). Moreover, we detected no nucleotide deletions, additions, or substitutions in the transgenes by Sanger sequencing. To independently address the issue of potential transgene loss, we also examined virus titers for founder and passage 33 viruses by comparing the numbers of PFU and fluorescent focus units (FFU). PFU would account for all infectious viruses (with and without RFP expression), while FFU would account only for viruses retaining their functional RFP transgene. We did not observe any significant changes in the FFU/PFU ratios for founder and passage 33 viruses indicating that the passage 33 viruses have not lost RFP transgene sequences (Fig. 2B). Together, our data demonstrate long-term stability of VSV recombinants carrying RFP-p53 transgenes after extended replication of tested viruses in either permissive or moderately resistant PDAC cell lines.

Figure 2C summarizes all genome alterations in viruses detected by Sanger sequencing. No mutations were detected in the VSV regions of N, M, p53, or RFP or any intergenic regions of the viral genome. The absence of any novel mutations in VSV-M after 33 passages is particularly important, indicating the stability of M-ΔM51 as an oncolytic virus attenuator. Of the passage 33 viruses that were passaged on the cell line MIA PaCa-2, one missense mutation, E860D, only partially present in passage 33 viral population (data not shown), was detected in the L protein coding region of VSV-p53wt (MIA PaCa-2). This mutation was not present in any other virus. As we expected, SUIT-2-passaged viruses acquired more mutations than the MIA PaCa-2-passaged viruses, likely because of the stronger selective pressures in SUIT-2 cells. VSV-p53wt (SUIT-2) had a total of 3 nucleotide (nt) substitutions: 2 missense mutations in VSV-G and one silent mutation in VSV-L. VSV-p53-CC (SUIT-2) had a total of 5 nt substitutions: 3 missense mutations in VSV-G, 1 silent mutation in VSV-P, and 1 silent mutation in VSV-L (Fig. 2C).

**FIG 3:**
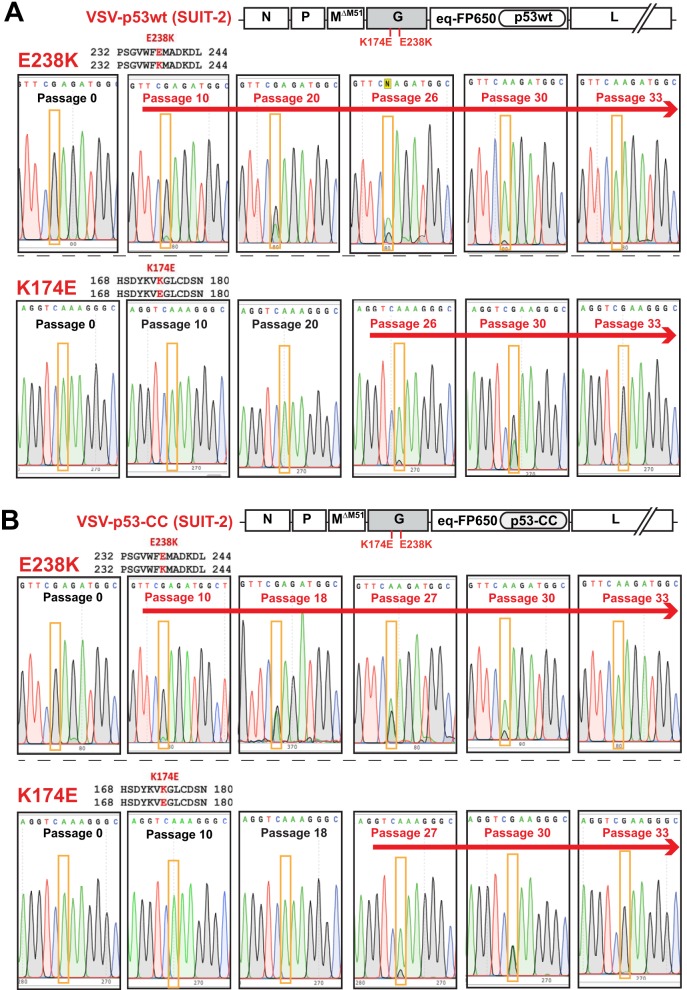
The chronological order of the appearance of VSV-G mutations E238K and K174E during passaging of VSV-p53wt (A) and VSV-p53-CC (B) on SUIT-2 cells. Supernatants containing viral particles for the shown passages were used to isolate viral genomic RNA, which was reversed transcribed into cDNA using random hexamers, and cDNA was PCR amplified and sequenced using VSV-specific primers. The nucleotide substitutions are highlighted in orange boxes, and the presence of either mutation is indicated by a red arrow above the sequences. The amino acid numbering starts from the first amino acid of the mature VSV-G and does not include the 16-aa N-terminal signal peptide.

Surprisingly, both of the SUIT-2-passaged viruses acquired 2 identical missense mutations in VSV-G at aa positions 174 (K174E, A→G substitution) and 238 (E238K, G→A substitution) (Fig. 2C). To see at what point these mutations occurred during viral passaging, we sequenced VSV-G of each virus at intermittent passages. Figure 3 shows that in both VSV-p53wt (SUIT-2) and VSV-p53-CC (SUIT-2), E238K appeared first around passage 10, followed by K174E that first appeared around passage 26 in VSV-p53wt (SUIT-2) and passage 27 in VSV-p53-CC (SUIT-2). Interestingly, only after K174E became dominant in both viruses (around passage 30), E238K quickly reached fixation (complete sweep) (Fig. 3). Also, while the E238K mutation was slowly replacing the WT position between passages 10 and 33, the K174E change reached fixation (complete sweep) surprisingly quickly, in just several passages after appearing first around passage 27.

**FIG 4:**
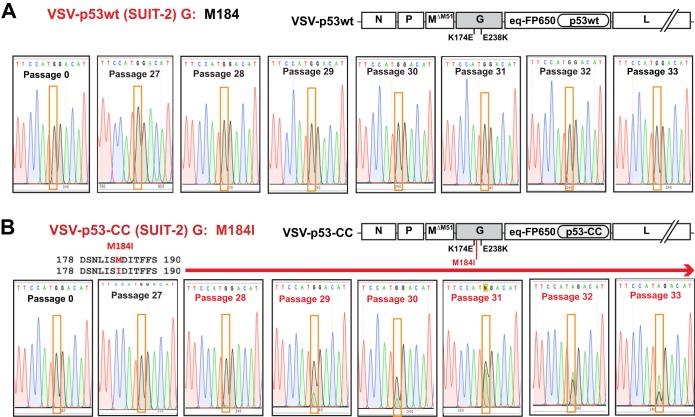
The chronological order of the appearance of VSV-G mutation M184I, which was found in VSV-p53-CC (SUIT-2), but not in VSV-p53wt (SUIT-2). Supernatants containing viral particles for the shown passages were used to isolate viral genomic RNA, which was reversed transcribed into cDNA using random hexamers, and cDNA was PCR amplified and sequenced using VSV-specific primers. The nucleotide substitutions are highlighted in orange boxes, and the presence of the M184I mutation is indicated by a red arrow above the sequences.

Compared to VSV-p53wt (SUIT-2), VSV-p53-CC (SUIT-2) obtained another mutation in VSV-G, M184I (G→A substitution) (Fig. 2C and Fig. 4). Although M184I has never completely replaced the WT position in the viral population of passage 33 VSV-p53-CC (SUIT-2), it was fixing in the population surprisingly quickly, first appearing at passage 28 and becoming prevalent by passage 33 (Fig. 4).

### SUIT-2-passaged viruses show an improved replication in PDAC cells, while retaining their oncoselectivity.

To determine whether the mutations in passaged viruses altered VSV abilities to replicate in PDAC or nonmalignant cells, virus replication kinetic assays were conducted to compare the founder viruses to the passage 33 viruses. In addition to MIA PaCa-2 and SUIT-2, we tested another human PDAC cell line, AsPC-1, which has a similar phenotype to SUIT-2 in terms of moderate resistance to VSV and inducible type I IFN signaling (37, 41). In addition, we tested the viruses in BHK-21 cells, which are highly permissive to VSV and many other viruses, at least in part due to their defective antiviral responses (67, 68). To examine the possible loss of oncoselectivity of the passaged viruses as a result of the acquired mutations, we also compared the viruses in the nonmalignant human pancreatic duct epithelial (HPDE) cell line (69) and the primary human fibroblast cell lines AG0159 and AG08498.

**FIG 5:**
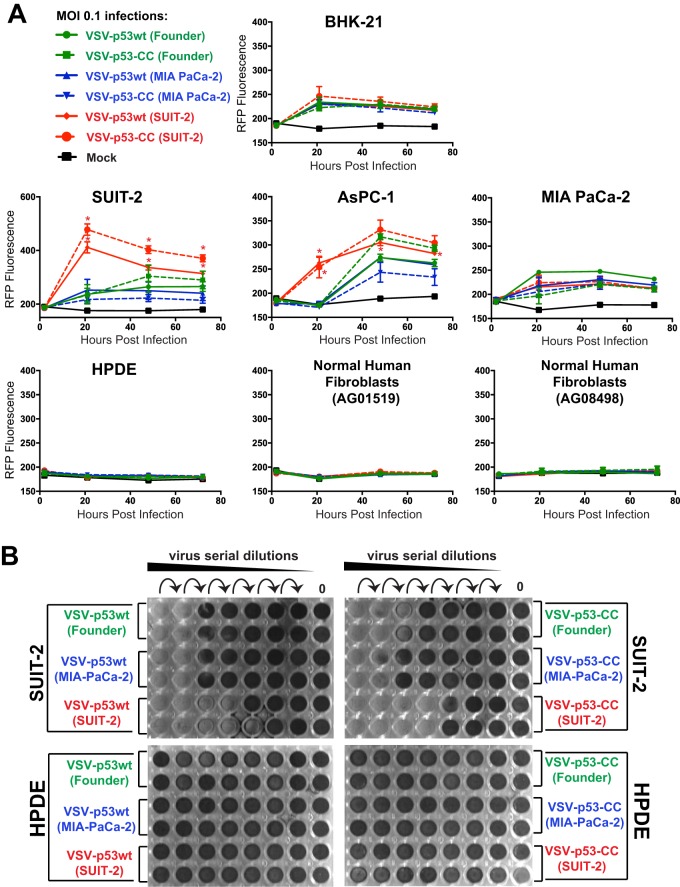
Viral replication kinetics of the founder and passage 33 viruses in different cell lines. (A) Cell lines were either mock treated or infected with a virus at an MOI of 0.1 PFU/cell (MOI calculated based on virus titration on BHK-21 cells). The level of VSV-encoded RFP fluorescence was measured over time from 1 h p.i. to 72 h p.i. The figure presents data representative of results from 2 independent experiments. The data points and error bars shown represent the means and SD of the means, respectively. *, *P* < 0.05. Results were analyzed to determine significance using two-way ANOVA with a Tukey posttest at a 95% confidence interval for comparison between each condition. If no error bars appear, the error is too small to appear on the graph. (B) Crystal violet cytotoxicity assay. The first well (on the left of each plate) was infected at an MOI of 0.15 (PFU calculated based on virus titration on BHK-21 cells), and then 6-fold serial dilutions were used to infect different cell lines in a 96-well format. Each cell line was also mock treated (control). Cells were stained with crystal violet solution (2% crystal violet in methanol) at 72 h p.i. to detect cytotoxicity caused by viruses, and unstained wells represent those in which total cell lysis had occurred. The figure shown is representative of results from at least 2 independent experiments.

Based on the determined virus titers on BHK-21 cells, different cell lines were infected at an MOI of 0.1, and VSV-encoded RFP fluorescence was measured at 1, 21, 48, and 72 h p.i. (Fig. 5A). As shown in previous studies, due to its downstream position between VSV-G and VSV-L, virus-encoded reporter expression can be used to measure virus replication levels as it could be detected only if the virus genome is replicated (70). The experiment showed that while all tested viruses showed similar levels of replication in BHK-21 cells, both VSV-p53wt (SUIT-2) and VSV-p53-CC (SUIT-2) replicated better in SUIT-2 cells, compared to founder viruses, especially at 21 h p.i. Importantly, both VSV-p53wt (SUIT-2) and VSV-p53-CC (SUIT-2) replicated better not only in SUIT-2 cells, but also in AsPC-1 cells, and they retained the abilities of founder viruses to replicate in MIA PaCa-2 cells, indicating that experimental evolution of viruses in SUIT-2 cells widened the range of PDAC cells permissive to VSV.

While SUIT-2-passaged viruses show an improved ability to replicate in the SUIT-2 and AsPC-1 cell lines, they also show a retention of oncoselectivity, as none of the tested viruses showed detectable replication in HPDE cells or either of the tested primary human fibroblast cell lines (Fig. 5A). In agreement with virus replication kinetics assay (Fig. 5A), crystal violet cell cytotoxicity assay showed improved cell killing for SUIT-2-passaged viruses in SUIT-2 cells, but no cell killing in HPDE cells (Fig. 5B).

### K174E is required for improved replication of VSV experimentally evolved in SUIT-2 cells.

**FIG 6:**
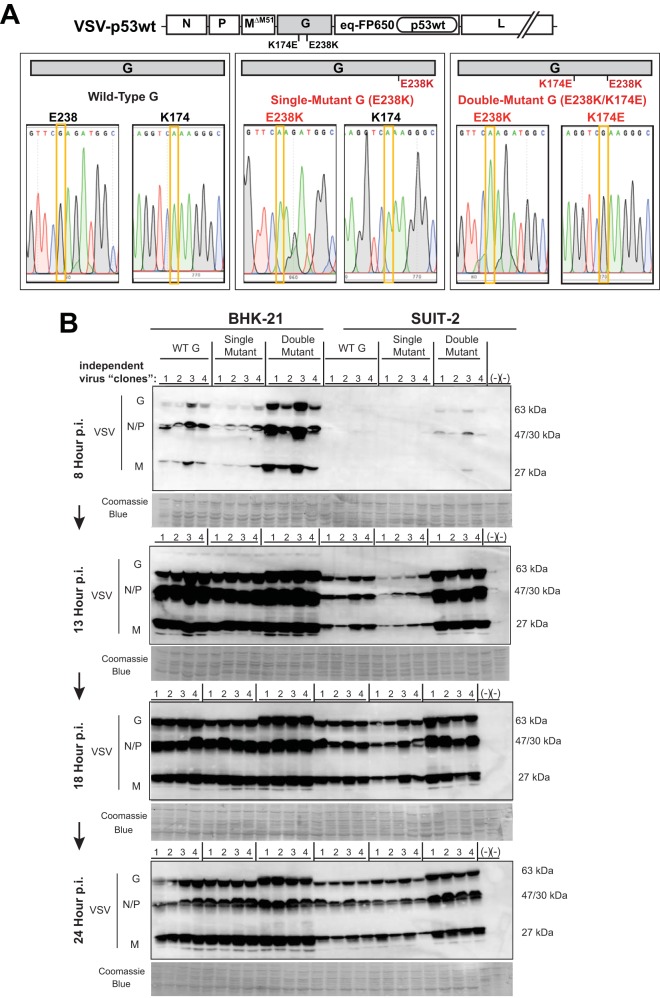
(A) Generation of VSV-p53wt viruses with WT, single mutant (E238K), and double mutant (K174E E238K) VSV-G. Eight independent plaque-isolated VSV-p53wt viruses were obtained by serial dilution of passage 20 (4 WT VSV-G and 4 single mutant E238K VSV-G) as well as 4 passage 33 (double mutant K174E E238K VSV-G) plaque-isolated viruses were generated from virus stocks until only 1 FFU was microscopically observed, and then each virus that originated from a single FFU was amplified in BHK-21 cells. Supernatants containing viral particles for the shown passages were used to isolate viral genomic RNA, which was reversed transcribed into cDNA using random hexamers. This cDNA was PCR amplified using VSV-specific primers, and the VSV-G regions containing aa positions 238 and 174 were sequenced. The nucleotide substitutions are highlighted in yellow boxes, and the presence of either mutation is indicated by a red arrow above the sequences. (B) BHK-21 and SUIT-2 cell monolayers were incubated with each virus at an MOI of 0.1 (MOI calculated based on virus titration on BHK-21 cells). Protein isolates were analyzed at 8, 13, 18, and 24 h p.i. and analyzed by Western blotting for expression of VSV proteins (G, N/P, and M). Lane numbers are indicated above membranes. Equal loading is indicated by Coomassie blue. Protein sizes are indicated on the side in kDa.

As both VSV-p53wt (SUIT-2) and VSV-p53-CC (SUIT-2) obtained the same 2 mutations in VSV-G (first E238K, then later K174E) (Fig. 2 and 3), we wanted to determine whether E238K alone or both mutations were required for the observed similar phenotypes of these SUIT-2-passaged viruses. To address this question, SUIT-2 passage 20 of VSV-p53wt (to isolate viruses with no mutations or only E238K) and SUIT-2 passage 33 of VSV-p53wt (to isolate viruses with both E238K and K174E mutations) were serially diluted until only 1 FFU was microscopically observed in a tissue culture well, and then each virus originated from a single FFU was amplified in BHK-21 cells, and the viral genome was sequenced to verify VSV-G sequence. Using this approach, we obtained 12 independent VSV-p53wt-based viruses, each of which originated from a single FFU (hereinafter referred to as “independent virus clones”), 4 with no mutations in G (WT VSV-G), 4 with only the E238K mutation (“single mutant”), and 4 with both E238K and K174E mutations (“double mutant”) (Fig. 6). No virus clones with only the K174E mutation were evaluated in this study as that mutation was only present together with the E238K mutation. Based on the determined virus titers on BHK-21 cells, BHK-21 and SUIT-2 cells were infected at an MOI of 0.1, and replication of these “independent virus clones” was examined by Western blotting by analyzing accumulation of viral proteins at 8, 13, 18, and 24 h p.i. As shown in Fig. 6, no stimulation of viral replication was observed for any of the 4 single mutants (G-E238K) in either BHK-21 or SUIT-2 cells, while clear improvement in viral replication can be seen for all double mutants at all tested time points in SUIT-2 cells and at earlier time points (especially at 8 h p.i.) in BHK-21 cells. These data indicate that the second mutation, K174E, was required for improved replication of VSV experimentally evolved in SUIT-2 cells.

### Acquired G mutations do not evade antiviral responses, but stimulate VSV replication at least in part due to improved virus attachment to SUIT-2 cells.

**FIG 7:**
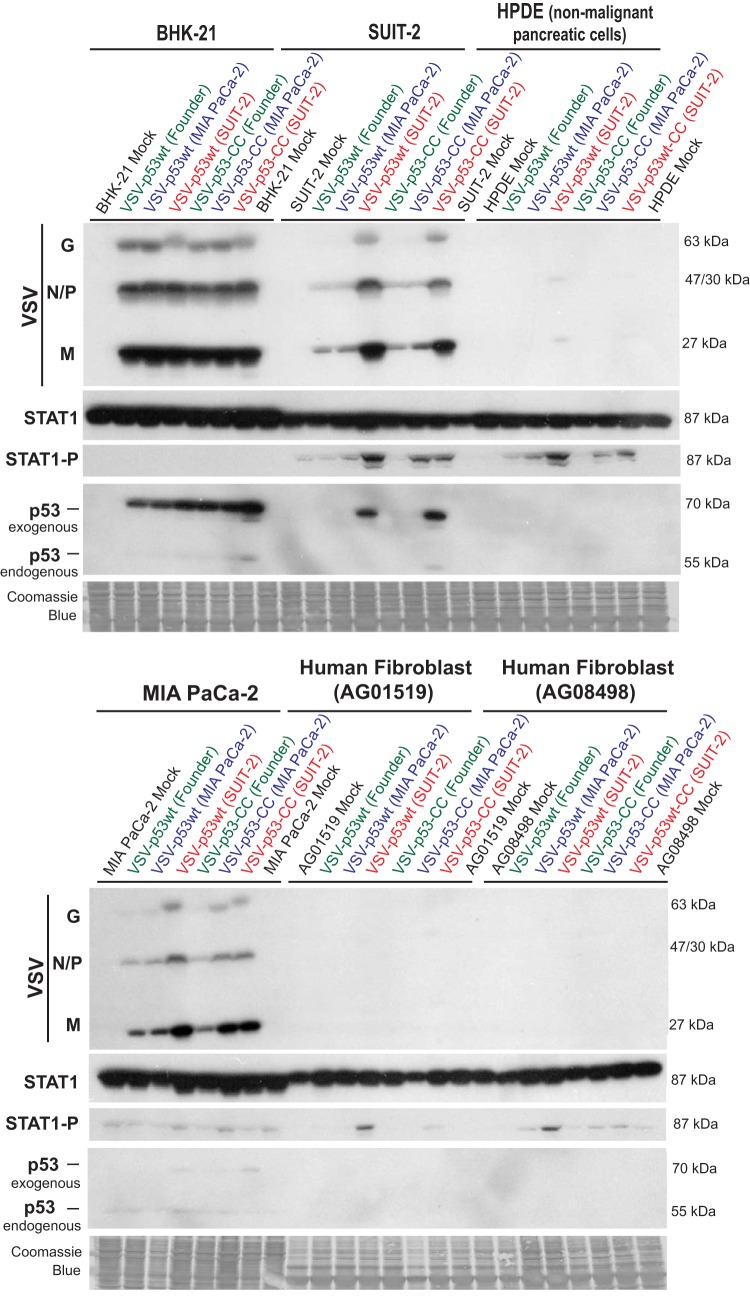
Replication of the founder and passage 33 viruses in different cell lines. Cell monolayers of different cell lines were incubated with VSV-p53wt (Founder), VSV-p53-CC (Founder), VSV-p53wt (MIA PaCa-2), VSV-p53-CC (MIA PaCa-2), VSV-p53wt (SUIT-2), and VSV-p53-CC (SUIT-2) at an MOI of 0.1 (MOI calculated based on virus titration on BHK-21 cells). Protein was isolated at 13 h p.i. and analyzed by Western blotting for total STAT1, phospho-STAT1 (STAT1-P), endogenous p53 (cell encoded), exogenous p53 (virus-encoded RFP-p53 fusion), and VSV proteins (N, P, M, and G). The observed size difference between endogenous p53 (around 53-kDa band) and exogenous p53 (around 70-kDa band) is due to the fact that N terminus of VSV-encoded p53 is fused to the C terminus of RFP in both VSV-p53wt and VSV-p53-CC viruses. Equal loading indicated by Coomassie blue. Protein sizes are indicated on the side in kDa.

Our previous analysis of permissive and resistant PDAC cell lines identified at least 2 mechanisms responsible for resistance of SUIT-2 cells to VSV. First, SUIT-2 cells are able to induce a functional type I IFN response to VSV (37, 41, 66). Second, we observed that, compared to MIA PaCa-2 and some other tested PDAC cell lines, VSV attaches less efficiently to SUIT-2 cells (44). Therefore, we hypothesized that extensive passaging of VSV on SUIT-2 cells selected for spontaneous VSV mutants via the following mechanisms: (i) an improved ability to evade type I IFN signaling and/or (ii) improved attachment to SUIT-2 cells. If the first hypothesis is correct, we would expect to see an increase in VSV replication accompanied by a decrease in antiviral signaling when SUIT-2 cells are infected with the evolved viruses. To test this hypothesis, we infected SUIT-2 and other cell lines shown in Fig. 7 with different viruses at an MOI of 0.1, and total protein was isolated at 13 h p.i. and analyzed by Western blotting for accumulation of VSV-encoded proteins as well as total STAT1 and phosphorylated STAT1 (STAT1-P) levels as a marker of type I IFN signaling induction (Fig. 7). In agreement with our data in Fig. 5 and 6, VSV-p53wt (SUIT-2) and VSV-p53-CC (SUIT-2) showed an increased ability to replicate on SUIT-2 cells (Fig. 7). Interestingly, these 2 viruses also showed a slightly increased ability to replicate in MIA PaCa-2 cells, which are defective in type I IFN signaling. We did not detect significant differences in viral replication levels in BHK-21 cells at 13 h p.i., which is consistent with the data shown in Fig. 6, where the differences in BHK-21 cells were seen mainly at an earlier time point (8 h p.i.). No significant viral replication was detected in the nonmalignant HPDE pancreatic ductal cell line HPDE and human primary fibroblast cell lines AG0159 and AG08498, confirming retained oncoselectivity of the evolved viruses (Fig. 7). Importantly, although viral infections did not significantly alter total STAT1 levels, for both of the SUIT-2-passaged viruses in the SUIT-2 and HPDE cell lines, as well as both of the fibroblast cell lines, there was an increase, rather than decrease, in STAT1 phosphorylation in VSV-p53-CC (SUIT-2)- and VSV-p53wt (SUIT-2)-infected cells (Fig. 7). This result shows that the improved replication of the evolved viruses was not due to their acquired abilities to evade innate antiviral responses. (In such a case, we would see inhibition rather than stimulation of STAT1 phosphorylation.) The observed increased STAT1 phosphorylation was likely a result of the higher number of cells infected with SUIT-2-passaged viruses, which produce collectively a stronger antiviral response.

**FIG 8:**
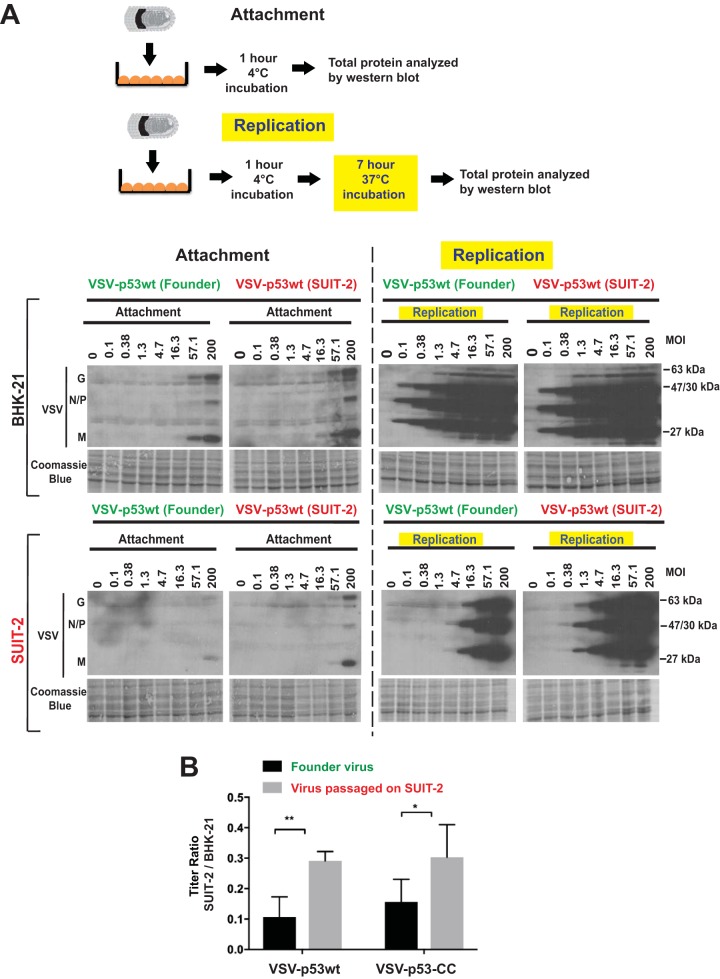
Comparison of VSV attachment and replication in BHK-21 and SUIT-2 cells. (A) For VSV attachment to cells in monolayers, cells were incubated with purified VSV-p53wt (Founder) or VSV-p53wt (SUIT-2). Cell monolayers were incubated with either virus at different MOI (calculated based on virus titration on BHK-21 cells) for 1 h at 4°C (attachment assay) only or for 1 h at 4°C and then an additional 7 h at 37°C (replication assay). Protein was isolated and analyzed by Western blotting. Coomassie blue stain was used to indicate equal loading of samples. Protein sizes are indicated on the side in kDa. (B) Virus titers on both SUIT-2 and BHK-21 cells compared for VSV-p53wt (Founder) compared to VSV-p53wt (SUIT-2) as well as VSV-p53-CC (Founder) compared to VSV-p53-CC (SUIT-2). Data bars and error bars represent the means and standard deviations (SD) of the means, respectively. Data represent the means and SD of the means. Conditions were compared using unpaired t-tests of at least 3 repeated experiments. *, *P* < 0.05; **, *P* < 0.01.

Since both VSV-p53wt (SUIT-2) and VSV-p53-CC (SUIT-2) mutations were located in VSV-G, a region that plays an important role in viral attachment, and because our previous studies have shown that VSV does not attach to SUIT-2 cells as well as to some other PDAC cell lines, we also compared the ability of the founder and SUIT-2-passaged viruses to attach to SUIT-2 cells. To examine virus attachment, BHK-21 or SUIT-2 cells were incubated at various MOIs with purified VSV-p53wt (Founder) or VSV-p53wt (SUIT-2) at 4°C for 1 h, and the cells were extensively washed to remove any unbound virus and analyzed by Western blotting for virus proteins bound to cells (attachment assay in Fig. 8). At 4°C, the viral particles can only attach to the outside of cells and not enter them. A duplicate set of cells was treated the same way (incubated with virus at 4°C for 1 h and then extensively washed), but then incubated for 7 more h at 37°C before protein was isolated to examine virus replication (replication assay in Fig. 8). In BHK-21 cells, there was only a minor difference in virus attachment or replication between VSV-p53wt (Founder) or VSV-p53wt (SUIT-2) viruses. However, in SUIT-2 cells, VSV-p53wt (SUIT-2) was able to attach much more efficiently (about 3-fold better based on serial dilutions of viruses) than VSV-p53wt (Founder), and VSV-p53wt (SUIT-2) replication of the passage 33 virus was also higher in SUIT-2 cells. Interestingly, we observed about 3-fold improvement in virus attachment, but about 10-fold enhancement in virus replication for the SUIT-2-passaged viruses in SUIT-2 cells. While it is possible that the 3-fold improvement in virus attachment was solely responsible for even stronger enhancement in virus replication due to the exponential growth rate of virus replication, we cannot rule out that E238K and K174E VSV-G mutations also improve other steps of the virus replication cycle. Future studies will test these possibilities.

These data suggest that the SUIT-2-passaged viruses were selected to attach to SUIT-2 cells more efficiently, which could improve new infection efficacy and explain at least in part the observed improvement in viral replication of SUIT-2-passaged viruses. To test whether SUIT-2-passaged viruses could initiate infections more efficiently, compared to founder viruses, titers of serial dilutions of each virus were determined on BHK-21 and SUIT-2 cells, and the ratios of virus titers on SUIT-2 cells to those on BHK-21 cells were calculated. As shown in Fig. 8B, VSV-p53wt (SUIT-2) and VSV-p53-CC (SUIT-2) improved their abilities to initiate infections on SUIT-2 cells by about 3-fold, which is consistent with our data on relative attachment efficiency of founder and passaged viruses (Fig. 8A).

**FIG 9:**
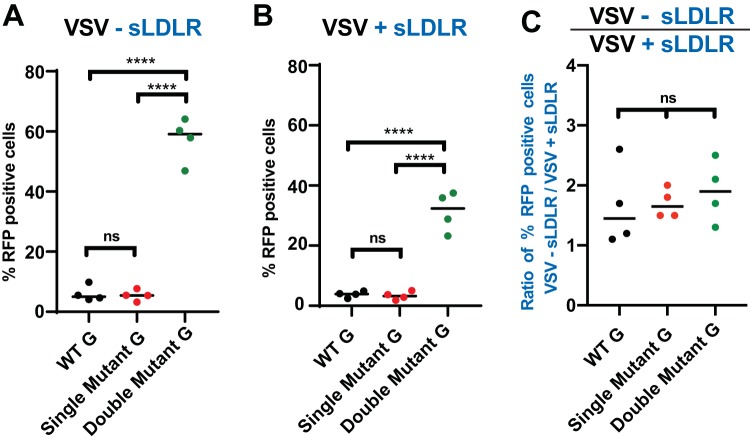
Effect of sLDLR on infectivity of VSV-p53wt viruses with WT, single mutant (E238K), and double mutant (K174E E238K) VSV-G. Four independent plaque-isolated VSV-p53wt viruses were obtained by serially diluting passage 20 (WT and single mutant E238K VSV-G) or passage 33 (double mutant K174E E238K VSV-G) virus stocks until only 1 FFU was microscopically observed, then each virus that originated from a single FFU was amplified in BHK-21 cells. Virus dilutions at an MOI of 0.1 were incubated without sLDLR (A) or with 1 μg/ml sLDLR (B) for 30 min at 37°C before being used in SUIT-2 infection (calculated based on virus titration on BHK-21 cells). After a 30-min infection, virus-containing medium was removed and fresh medium was added. Thirteen hours p.i., RFP-positive cells were counted using a Nexcelom Vision Image cytometer. The data presented are representative of 2 independent experiments. The data points and error bars shown represent the means and SD of the means, respectively. Results were analyzed to determine significance using one-way ANOVA with a Tukey’s posttest at a 95% confidence interval for comparison between each condition. ****, *P* < 0.0001; ns, nonsignificant. (C) Percentage of RFP^+^ infected cells without sLDLR divided by the percentage of RFP-infected cells in the presence of sLDLR compared to determine the relative inhibition of sLDLR on WT, single mutant, and double mutant VSVs.

LDLR and LDLR family members have been shown to serve as receptors for VSV (3 – 6). As VSV-G is responsible for VSV attachment to host cells and we observed an improved attachment of SUIT-2-passaged viruses to SUIT-2 cells (Fig. 8A), we wanted to examine the abilities of the evolved viruses to attach to LDLR. Mutations in VSV-G could improve VSV’s ability to interact with LDLR or, rather, utilize an alternative receptor. The affinity of VSV for LDLR was examined using soluble LDLR (sLDLR), which neutralizes VSV virions and inhibits viral infectivity. To test if sLDLR could inhibit infectivity of “independent virus clones” (WT-G, single mutant, or double mutant) of VSV-p53wt, the same number of infectious particles corresponding to an infection at an MOI of 0.1 on BHK-21 cells were incubated with or without sLDLR *in situ*, and the SUIT-2 cells were incubated with these VSV ± sLDLR combinations for additional 30 min, washed to remove any unattached virus and sLDLR, and then incubated for an additional 12 h. Cells were then trypsinized and analyzed for the percentage of VSV-infected cells (RFP-positive cells). In agreement with Fig. 6 data, the presence of both VSV-G mutations E238K and K174E in double mutants resulted in a dramatic increase in the percentage of VSV-infected cells in the absence of sLDLR (Fig. 9A). In the presence of sLDLR (Fig. 9B), we observed a significantly lower percentage of VSV-infected cells for all tested viruses (Fig. 9B). However, we did not observe statistically significant differences between viruses with regard to the inhibiting effect of sLDLR on viral infectivity when we compared the ratios of VSV-infected cells in the absence and presence of sLDLR, which were close to 2 under our experimental conditions (Fig. 9C). These data suggest that VSV is still able to attach to and infect SUIT-2 cells through an interaction with LDLR.

**FIG 10:**
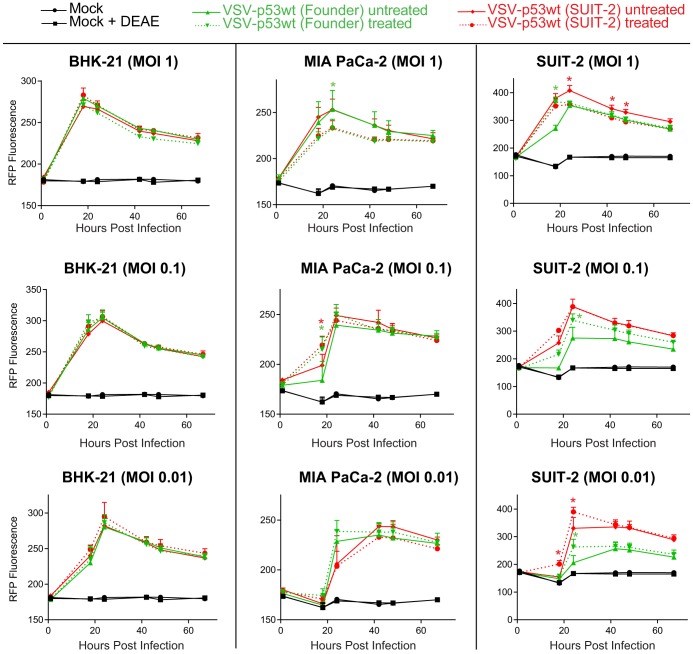
Effect of polycation DEAE-dextran on VSV infection in different cell lines. Cells were pretreated with 10 μg/ml DEAE-dextran or mock treated for 30 min and then infected with the indicated virus at an MOI of either 1, 0.1, or 0.01 (MOI calculated based on virus titration on BHK-21 cells) for 1 h at 37°C. Medium containing virus plus DEAE-dextran was removed after the 1-h infection period, and fresh DMEM with 5% FBS was added to the cells. The level of VSV-encoded RFP fluorescence was measured over the course of 68 h. The figure presents data representative of results from 3 independent experiments. The data points and error bars shown represent the means and SD of the means, respectively. Results were analyzed to determine significance using two-way ANOVA with a Tukey’s posttest at a 95% confidence interval for comparison between each condition. *, *P* < 0.05. If no error bars appear, the error is too small to appear on the graph.

We have previously shown that infectivity of VSV-ΔM51 in several resistant PDAC cell lines, including SUIT-2, was dramatically improved when cells were treated with the polycations DEAE-dextran or Polybrene (44). Although the exact mechanism of polycation-mediated improvement of virion attachment is not clear and several alternative mechanisms, including charge shielding and virus aggregation, have been proposed (71), it is believed that polycations help the initial nonspecific anchoring of virus particles to cell surface, which facilitates their further association with specific receptors (such as LDLR and LDLR family members for VSV) (72 – 76). As our data suggest that the VSV-G mutations E238K and K174E did not dramatically change VSV-G affinity for LDLR, we decided to test a hypothesis that E238K and K174E mutations improve the efficacy of this initial nonspecific VSV binding to target cells. In such a case, the efficient infection of evolved viruses would be less dependent on polycation treatment, compared to the founder viruses. To test this hypothesis, we infected BHK-21, MIA PaCa-2, and SUIT-2 cells with the founder viruses and the SUIT-2-passaged viruses at various MOIs in the presence or absence of DEAE-dextran and analyzed virus replication kinetics by measuring VSV-encoded RFP fluorescence over time. In agreement with our previous study using VSV-ΔM51 (44), we did not observe any significant positive effect of DEAE-dextran on replication of any tested viruses in the highly permissive BHK-21 and MIA PaCa-2 cell lines (Fig. 10). On the other hand, in agreement with the same study (44), DEAE-dextran treatment strongly improved infectivity of VSV-p53 (Founder) at all tested MOIs in SUIT-2 cells (Fig. 10). In contrast, DEAE-dextran treatment had a rather small positive effect on VSV-p53 (SUIT-2) infection at only the lowest MOI tested (MOI of 0.01), and no effect was observed at an MOI of 0.1. Moreover, DEAE-dextran treatment actually inhibited VSV-p53 (SUIT-2) at an MOI of 1 (Fig. 10). In general, these data indicate that VSV-G mutations K174E and E238K make the evolved viruses less dependent on polycations for efficient infection of resistant cell lines, such as SUIT-2, suggesting that the evolved viruses have an improved nonspecific attachment to target cells.

## DISCUSSION

In this study, using a directed viral evolution approach, we generated novel oncolytic VSVs that show improved replication in virus-resistant PDAC cells. Both SUIT-2-passaged VSV-p53wt and VSV-p53-CC showed improved replication in the human PDAC cell lines SUIT-2 and AsPC-1, which are moderately resistant to VSV, and they retained the abilities of founder viruses to replicate in MIA PaCa-2 cells, indicating that experimental evolution of viruses in SUIT-2 cells widened the range of PDAC cells permissive to VSV. Importantly, both evolved viruses remained highly attenuated in the nonmalignant human pancreatic duct epithelial cell line HPDE and the primary human fibroblast cell lines AG0159 and AG08498. Moreover, no mutations were found in M-ΔM51, and no deletions or mutations were found in the p53 or eqFP650 portions of virus-carried transgenes in any of the passaged viruses, demonstrating long-term genomic stability of complex VSV recombinants carrying large eqFP650-p53 transgenes.

Interestingly, both of the SUIT-2-adapted viruses passaged in parallel, VSV-p53wt (SUIT-2) and VSV-p53-CC (SUIT-2), acquired two identical missense mutations in VSV-G, E238K and K174E. The fact that both mutations (complete sweep) were acquired independently in two different viruses passaged in parallel suggests a strong fitness benefit to VSV in SUIT-2 cells from the introduction of these mutations. Interestingly, both viruses first obtained the E238K mutation before acquiring the K174E mutation. It is likely that the first mutation, E238K, provided a minor fitness benefit to both VSV-p53wt and VSV-p53-CC, founder viruses, but the observed major shift in virus replication required both mutations. It should be noted that the second mutation, K174E, was never identified alone in any of the viral passages and thus was not individually evaluated. VSV-p53-CC (SUIT-2) also showed a third missense mutation in VSV-G, M184I, but only in a portion of the passage 33 VSV-p53-CC population (not a complete sweep). Although it will be interesting to examine the role of this mutation in VSV replication in the future studies, it is unlikely that this particular mutation plays a critical role in the improved ability of this virus to replicate in SUIT-2 cells, as VSV-p53wt (SUIT-2) and VSV-p53-CC (SUIT-2) exhibited very similar phenotypes.

Although the VSV-G E238K K174E double mutant had never been described in the past, a previous study described VSV-G E238G (VSV mutants “G_6_” and “G_6R_”) and E238Q (VSV mutants “G_5_” and “G_5R_”) mutants (63). Most of these previously described VSV mutants had additional mutations in VSV-G, although one of the mutants, VSV-G_6R_, had only the single amino acid substitution E238G in VSV-G (63). The study showed that VSV-G_6R_ infection of L929 cells (mouse fibroblasts) produced higher levels of IFN-β compared to WT VSV, and those levels were similar to or even higher than those with the VSV-M51R mutant. Based on that result, the authors proposed that E238G mutation in VSV-G enhances type I interferon secretion and responses via some unclear mechanism not involving VSV-M (63). However, we propose another explanation for their observations: that E238G mutation may have resulted in an improved replication of VSV-G_6R_ virus in L929 cells, and the observed overall increase in IFN-β was due to the increase in the number of infected cells rather than the increased IFN-β production by each infected cell. In agreement with this hypothesis, no attenuation was observed for VSV-G_6R_ replication in L929 cells, which would be expected for a mutant in which replication enhances antiviral response (63). Our data show the increased STAT1 phosphorylation in cells infected with the SUIT-2-adapted viruses carrying the E238K K174E VSV-G double mutation, but we think that was due to their higher levels of replication, which we observed throughout this study, suggesting that these evolved viruses are not modulating antiviral signaling in each cell, but instead the higher number of infected cells (Fig. 9) produces collectively higher STAT1 phosphorylation levels.

Although we cannot exclude additional mechanisms of the improved VSV replication in the presence of E238K and K174E mutations in VSV-G, our data show that these mutations result in improved VSV attachment to SUIT-2 cells. We envision that during VSV passaging, when a virus passage was incubated with fresh cells for a 1-h period (after which the incubation medium containing unbound virus was aspirated and cells were washed with phosphate-buffered saline [PBS]), there was a selective pressure for VSV mutants capable of more efficient attachment to SUIT-2 cells.

**FIG 11:**
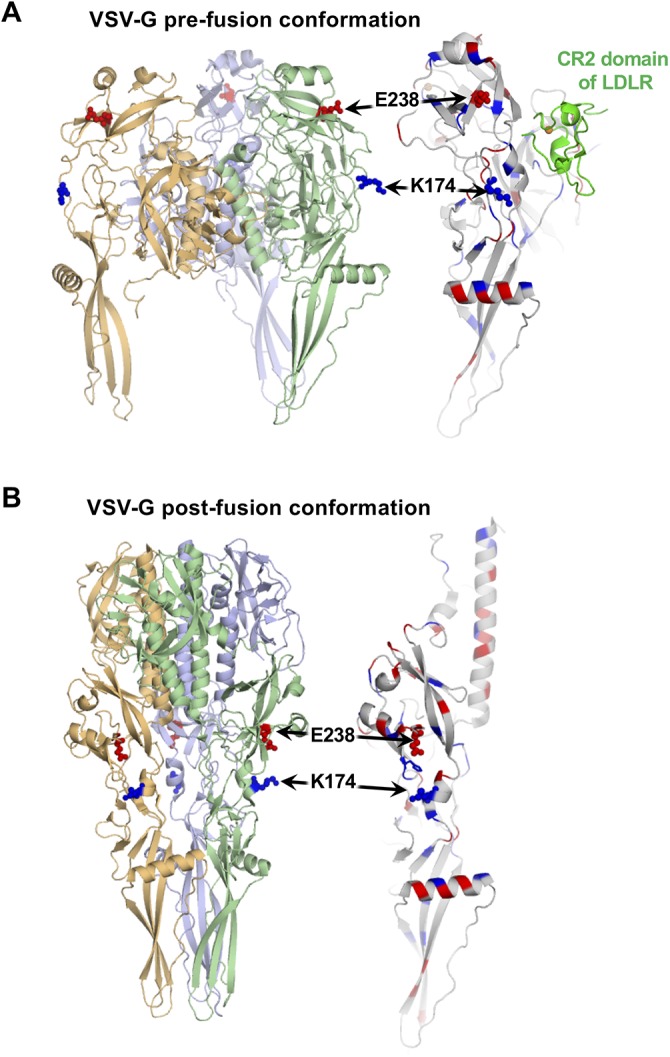
Locations of VSV-G E238K and K174E mutations on crystallographic structures of prefusion and postfusion states of the glycoprotein G. (A) Cartoon representation of the prefusion conformation of protein G bound to the cysteine-rich domain of LDLR. Positively and negatively charged residues are shown by blue and red, respectively. E238 and K174 are shown using sticks and spheres. E238 is surrounded by positively charged residues. Both E238 and K174 are positioned away from the binding interface of the VSV-G and LDLR CR2 region. (B) Cartoon representation of the low-pH, postfusion conformation of VSV-G trimer (left) and VSV-G monomer (right). Color coding is the same as in panel A. Both E238 and K174 are positioned away from the intermonomer interface in the protein VSV-G trimer (left). Histidine H226 is sandwiched by E238 and K174 (right).

It is unclear how the E238K and K174E mutations in VSV-G improve VSV attachment to PDAC cells. We analyzed the positions of E238K and K174E mutations using the crystallographic structures of prefusion conformations of VSV-G with and without LDLR (PDB code no. 5oyl, 5oy9, and 5i2s) and low-pH, postfusion conformations of VSV-G without LDLR (PDB code no. 2cmz) (6, 77, 78) (Fig. 11). As shown in Fig. 11, both mutations are located away from the interaction interface between VSV-G and the CR2 or CR3 (cysteine-rich) domains of LDLR. Both mutations are also located on the side of the protein opposite to the intermonomer interface in the postfusion trimer. Therefore, it is unlikely that they affect the trimerization of the glycoprotein G or its interaction with LDLR, at least with the CR2 or CR3 domains for which X-ray structures have been solved. In agreement with that, we did not detect any significant differences between WT and mutant G viruses with regard to the inhibiting effect of sLDLR on viral infectivity (Fig. 9C). Also, our previous study showed that, despite lower levels of VSV attachment to SUIT-2 cells, SUIT-2 expressed high levels of LDLR, suggesting no limitation of the surface receptor for VSV in this cell line (44). Together, these data suggest that VSV-G mutations did not dramatically alter the abilities of mutant VSV-G proteins to attach to and infect SUIT-2 cells through an interaction with LDLR.

Interestingly, our data indicate that the VSV-G mutations E238K and K174E make the evolved viruses less dependent on polycations for efficient infection of SUIT-2. Previous studies have suggested that efficient cell attachment of virions of many viruses, including VSV and VSV-G-pseudotyped viruses (79, 80), requires an initial nonspecific binding of virus particles to cell surface, followed by attachment of virus particles to their specific receptors, which is required for virus internalization (72 – 76). The initial nonspecific binding step can be dramatically enhanced by treating cells with polycations, such as DEAE-dextran or Polybrene (81 – 83). Importantly, we have previously shown that infectivity of VSV-ΔM51 in several resistant PDAC cell lines, including SUIT-2, was dramatically improved when cells were treated with the polycation DEAE-dextran or Polybrene (44). Although the exact mechanism of polycation-mediated improvement of virion attachment is not clear and several alternative mechanisms, including charge shielding and virus aggregation, have been proposed (71), the most widely accepted hypothesis is that polycations decrease electrostatic repulsion between negatively charged molecules on the surface of cells and many viruses, including VSV, and thus facilitate nonspecific binding of virus particles to cell surface (82 – 84). Our data indicate that the VSV-G mutations E238K and K174E make the evolved viruses less dependent on polycations—possibly by decreasing dissociation of virions at the initial step of attachment due to electrostatic repulsion between cell surface molecules and VSV. One possibility is that these mutations change the overall structure of VSV-G, which could significantly change charge distribution on the surface of VSV particles, possibly reducing the repulsion. These mutations could also reduce repulsion by posttranslational modifications of VSV-G: for example, VSV-G N-glycosylation that occurs through the N163 and N320 positions (85 – 92). The VGV-G N-glycosylation can dramatically affect viral infectivity, although the effect strongly depends on the target cell type and specific mode of VSV-G N-glycosylation (89, 90, 93). Moreover, it is believed that one of the major mechanisms of DEAE-dextran-mediated improvement of VSV infection is removal of repulsion between negatively charged molecules on the cell surface (such as anionic phospholipid sialic acid residues) and terminal sialic acid residues associated with those N-glycosylated VSV-G (89). Therefore, it is possible that E238K or K174E mutations could alter VSV-G N-glycosylation pattern or juxtaposition of sialic acid residues and thus decrease repulsion, thus making mutant viruses less dependent on DEAE-dextran for virus infection.

We cannot rule out that E238K and K174E VSV-G mutations could also improve other steps of the virus replication cycle. VSV-G is responsible for both viral attachment and entry into host cells, and it is possible one or both of these mutations improved not only attachment, but also virus entry into the infected cells. Interestingly, E238K and K174E mutations are located near the region that undergoes the major conformational rearrangement during the transition from the prefusion to postfusion state (6, 77, 78). E238 and K174 are about 24 Å apart in the prefusion conformation, but only about 10 Å apart in the postfusion conformation (Fig. 11). It is possible that these mutations play a role in this transition. Future studies will test these possibilities.

Each of the passaged viruses was fully sequenced and compared to its corresponding founder virus. This analysis revealed overall impressive resistance of complex oncolytic VSV recombinants to mutations as none of the passaged virus had any nucleotide changes in the noncoding regions or amino acid substitutions in the N, P, or M region. Importantly, no viruses had any secondary mutations in M-ΔM51. We have demonstrated that not only can VSV’s efficacy as on OV be improved, it can do so while retaining oncoselectivity. This retention of oncoselectivity was demonstrated on 3 separate nonmalignant cells lines: HPDE (nonmalignant human pancreatic ductal cells) and the primary human fibroblast lines AG0159 and AG08498.

Over the course of 33 viral passages, all viruses retained both the p53 (p53wt or p53-CC) and RFP sequences of the virus-carried transgenes. No transgenes in any of the viruses obtained any mutations, demonstrating long-term genomic stability of complex VSV recombinants carrying large transgenes, even after replication over an extended period of time (more than 768 h of continuous viral replication). To our knowledge, our study is the first to passage VSV carrying a transgene for 33 passages and exhibiting complete transgene retention and absence of any mutations in the transgenes. This result is surprising considering that neither p53 nor RFP expression presumably benefits VSV-ΔM51 replication in MIA PaCa-2 cells, although p53 expression stimulates VSV-ΔM51 replication in SUIT-2 cells via inhibition of antiviral signaling (37). RNA viruses are known to have high mutation rates due to a lack of proofreading activities by viral RNA-dependent RNA polymerase (46, 94). This lack of proofreading can result in the introduction of mutations in the viral genome or transgenic regions. Such mutations can have a detrimental effect on the expression of viral or recombinant proteins. For instance, in a study where VSV expressed recombinant CD4 protein, a single nucleotide deletion resulting in a frameshift mutation caused the loss of expression of the transgene that was seemingly stable after 26 passages (95). In the same study, another VSV recombinant carrying measles virus F protein lost the transgene after 1 passage.

We think several different factors contributed to the surprising transgenic stability of our tested viruses. First, while NNS RNA viruses, like any RNA viruses, are associated with a high mutation rate (46, 94), they show lower incidence of genetic recombination caused by polymerase slippage compared to positive-strand RNA viruses, because the viral genome in NNS RNA viruses is encapsidated at all times, as opposed to naked RNA genomes of positive-strand RNA viruses, where polymerase can disassociate with one strand of RNA and reassociate on another strand (96, 97). Second, the helical nucleocapsid and overall bullet shape of VSV can accommodate extra transgenes by adding length to the virus rather than being geometrically limited in icosahedral virions of some other viruses (98). Third, our transgene sequences were not fused to viral proteins, which could increase selective pressures to lose the transgene. Previously, an in-frame fusion of green fluorescent protein (GFP) to VSV-P, an essential viral protein, resulted in the loss of transgene expression within 20 passages, assumedly because the reporter gene resulted in reduced viral fitness (99). Fourth, our viruses had transgene sequences located between VSV-G and VSV-L genes. Although placing a transgene closer to the 3′ end of the viral genome can increase its expression, it also results in greater virus attenuation (47, 97, 100, 101), which increases the selective pressures to lose the transgene sequences. On the other hand, several studies have successfully inserted transgenes between the G and L proteins of both VSV and rabies virus (RABV) without diminishing viral replication or activity (47, 102 – 104). Finally, the observed stability of the RFP-p53 transgenes is likely due to the selective pressures associated with beneficial effect of p53 expression on VSV replication in PDAC cells. The fact that p53 transgene was stable in both cell lines after 33 passages suggests that p53 has at least a minor positive effect on VSV replication even in MIA PaCa-2 cells.

While this study was focused on the adaptation of oncolytic VSV recombinants to PDAC cells and VSV transgene stability after extended virus passaging, future experiments will compare the efficacy and safety of the founder and SUIT-2-passaged VSV-p53 viruses *in vivo*. With our current findings describing improved VSV-p53 viruses and demonstrating long-term genomic stability of complex VSV recombinants encoding p53 transgenes, all of these data support further clinical development of VSV-p53 OVs as safe therapeutics for cancer.

## MATERIALS AND METHODS

### Viruses and cell lines.

The recombinant viruses VSV-p53wt and VSV-p53-CC were previously engineered using the VSV-ΔM51 backbone and were previously described in detail (37). Purified virus was obtained exactly as described previously (105). Plaque-isolated viruses were obtained by isolating individual viral plaques, which were then amplified on BHK-21 cells. The baby hamster kidney BHK-21 fibroblast cell line (ATCC CCL-10) was used to grow viruses and to determine their titers. Viral titers for both viruses were determined by standard plaque assay on BHK-21 or SUIT-2 cells using an agar overlay and then calculated as PFU/ml or FFU/ml. To calculate PFU/ml, cells were fixed and stained with crystal violet, whereas to calculate FFU/ml, VSV-encoded RFP fluorescent foci were counted using fluorescence microscopy. The following human PDAC cell lines were used in this study: SUIT-2 (106), MIA PaCa-2 (ATCC CRL-1420), and AsPC-1 (ATCC CRL-1682). The human origin of all these PDAC cell lines was confirmed by partial sequencing of KRAS and actin, as well as genomic mutation profiling using Cancer Hotspot Panel v2 (Life Technologies) to analyze for 2800 Catalogue of Somatic Mutations in Cancer (COSMIC) mutations of 50 oncogenes and tumor suppressor genes (43). As expected, all PDAC cell lines had a mutation in KRAS, as it is typical for PDACs (42, 43). A nonmalignant human pancreatic duct epithelial (HPDE) cell line was previously generated by introduction of the E6 and E7 genes of human papillomavirus 16 into normal adult pancreas epithelium. HPDE retains a genotype similar to pancreatic duct epithelium, is nontumorigenic in nude mice, and has no cancer-associated mutations (69). HPDE was grown in keratinocyte-SFM (K-SFM; Gibco, 17005042) without serum. Normal untransformed human fibroblast AG08498 and AG01519 cells from foreskin of healthy donors were obtained from the Coriell Institute. MIA PaCa-2, SUIT-2, and AsPC-1cells were maintained in Dulbecco’s modified Eagle’s medium (DMEM [Cellgro, 10-013-CV]), while BHK-21, AG08498, and AG01519 cells were grown in modified Eagle’s medium (MEM [Cellgro, 10-010-CV]). All cell growth media (except for K-SFM, which was supplemented with manufacturer-provided human recombinant epidermal growth factor 1-53 [EGF 1-53] and bovine pituitary extract [BPE]) were supplemented with 10% fetal bovine serum (FBS [Gibco]), 4 mM l-glutamine, 900 U/ml penicillin, 900 μg/ml streptomycin, and 1% nonessential amino acids. MEM was additionally supplemented with 0.3% glucose (wt/vol). Cells were kept in a 5% CO_2_ atmosphere at 37°C. For all experiments, all cell lines were passaged no more than 15 times.

### Viral passaging.

MIA PaCa-2 and SUIT-2 cells were seeded into 35-mm-diameter dishes to be approximately 95% confluent in 24 h. Cells were washed once with PBS and incubated with viruses at an MOI of 0.1 PFU/ml (calculated based on virus titration on BHK-21 cells) in DMEM without fetal bovine serum (FBS) for 1 h at 37°C. After 1 h of incubation, medium containing unbound virus was aspirated, cells were washed with PBS, and fresh DMEM medium containing 5% FBS was added to the cells. After 24 h of incubation, dishes were checked under a microscope (Olympus IX70 fluorescence microscope) to ensure all cells were infected, which was detected by the presence of RFP signal, and that all cells were detached from the 35-mm dishes. The entire supernatant was collected at 24 h p.i. and centrifuged at 4,000 rpm at 4°C for 10 min to pellet cellular material. The virus containing supernatant was transferred to new tubes and stored at –80°C. Each collected viral passage was used for the subsequent viral passage.

### Viral replication kinetics assay and crystal violet cytotoxicity assay.

For all experiments, MOI was determined by determining the titer of viruses using standard plaque assays on BHK-21 cells in 24-well plates. For virus replication kinetics assays, cells were seeded in 96-well plates. Viral dilutions of all viruses were prepared in DMEM with 0% FBS and used to infect cells at an MOI of 0.1. Cells were washed once with PBS, and virus was added to cells, which were incubated at 37°C for 1 h. Virus-containing medium was aspirated, and fresh DMEM with 5% FBS was added to cells that were further maintained at 37°C for the duration of the experiment. Virus-encoded RFP fluorescence levels were measured following incubation at 1, 21, 48, and 72 h p.i. using a fluorescence multiwell plate reader. RFP fluorescence was read at the wavelength 590/645 nm. For crystal violet cytotoxicity assay on multiple different cell lines, in a 96-well format, the first well was infected at an MOI of 0.15 (PFU calculated based on virus titration on BHK-21 cells), and then 6-fold serial dilutions were used to infect different cell lines. Each cell line was also mock treated (control). Cells were stained with crystal violet solution (2% crystal violet in methanol) at 72 h p.i. to detect cytotoxicity caused by viruses, and unstained wells represent those in which total cell lysis had occurred.

### RNA isolation, cDNA generation, PCR amplification, and DNA sequence analysis.

RNA was isolated from 100 μl of virus-containing supernatant using the Quick-RNA MiniPrep kit (Zymo Research, R1031 and R1033). Five microliters of total RNA per reverse transcription reaction mixture and random hexamer primers were used with SMART-Scribe reverse transcriptase (TaKaRa Bio, ST0065) to generate cDNA. PCR was done on the generated cDNA using VSV- or transgene-specific primers. PCR products were electrophoresed on 1% agarose gels containing ethidium bromide in TBE buffer, and PCR products were cut from the agarose gel from which DNA was extracted following the DNA extraction kit protocol (Qiagen, 28706). In a microcentrifuge tube, Following the Eurofins Genomics instructions, DNA with a concentration between 20 and 60 ng/μl was combined with a single primer. The DNA and primer combinations were sent to Eurofins Genomics for Sanger sequencing. As per the Eurofins Genomics sequencing algorithm, any base pair that obtained a Phred quality score of 20 or lower was marked as nonspecific (N). A Phred quality score of 20 or lower indicates a base call accuracy between 90 and 99%. All sequencing results were analyzed with SnapGene 4.3 software.

### Western blot analysis.

Cells were seeded into 12-well plates to be approximately 95% confluent after 24 h. Medium was removed, and cells were washed once with PBS. Virus was then added at an MOI 0.1 (calculated based on virus titration on BHK-21 cells) in 0% FBS medium and incubated for 1 h at 37°C. After 1 h of incubation, the virus-containing medium was removed, and 5% FBS medium was added to the cells. Cells were lysed and total protein was isolated 13 h p.i. using buffer exactly as described previously (66). Total protein was separated by electrophoresis on 10% SDS-PAGE gels and electroblotted onto polyvinylidene difluoride (PVDF) membranes. Membranes were blocked by using 5% nonfat powdered milk in TBS-T (0.5 M NaCl, 20 mM Tris [pH 7.5], 0.1% Tween 20) overnight at 4°C or for 1 h at room temperature. Membranes were incubated with a 1:5,000 dilution of rabbit polyclonal anti-VSV antibodies (raised against VSV virions), a 1:1,000 dilution of rabbit anti-STAT1 total (Cell Signaling, 14994T, clone D1K9Y), a 1:1,000 dilution of anti STAT1-phospho (Cell Signaling, D4A7), or a 1:5,000 dilution of anti-p53 (Cell Signaling, 1C12) in TBS-T with 5% milk with 0.02% sodium azide. For detection of horseradish peroxidase (HRP)-conjugated secondary antibodies, anti-rabbit (Jackson ImmunoResearch, 111-035-003) and anti-mouse (Jackson ImmunoResearch, 115-035-003) IgG, the Amersham ECL Western blotting detection kit was used. Alternatively, StarBright Blue 700 goat anti-mouse (Bio-Rad, 12004158) and anti-rabbit (Bio-Rad, 12004161) IgG fluorescent secondary antibodies at 1:5,000 dilutions were used for fluorescent Western blotting detection using the ChemiDoc MP imaging system from Bio-Rad. To verify total protein in each loaded sample, the membranes were stained with Coomassie blue.

### Virion attachment assay.

To assess VSV attachment to the cell monolayer, cells were seeded into a 12-well plate so that confluence was approximately 100% the next day. Medium was then removed, and cells were washed one time with PBS. Cells were place on ice approximately 5 min prior to virus infection to cool cells. Virus in DMEM (SUIT-2) or MEM (BHK-21) with 0% FBS was added to cells on ice, and cells were incubated for 1 h at 4°C. After incubation, virus-containing medium was aspirated, and wells were washed 3 times with PBS to remove any unbound virus. Samples then either had protein isolated immediately, as previously described (to examine attachment), or were incubated for an additional 7 h at 37°C (to examine VSV replication) and then had total protein isolated. Total protein was analyzed by Western blotting as described above. Membranes were initially blocked in 5% nonfat milk in TBS-T. Membranes were then incubated with a 1:5,000 dilution of rabbit polyclonal anti-VSV antibodies (raised against VSV virions) in TBS-T with 5% milk followed by a 1:10,000 dilution of anti-rabbit secondary antibodies. To verify total protein in each sample, loaded membranes were stained with Coomassie blue.

### Inhibition of VSV infection with soluble LDLR.

To analyze the effect of sLDLR on VSV, the E238K single mutant, and the E238K K174E Double Mutant, cells were seeded into 12-well plates so that they were 95% confluent after 24 h. A virus dilution at an MOI of 0.1 without sLDLR or with 1 μg/ml of sLDLR (R&D Systems, catalog no. 2148-LD-025) was incubated for 30 min at 37°C. Medium was aspirated from the cells, which were then washed once with PBS. Virus dilutions incubated with or without sLDLR for 30 min were added to cells and incubated for 30 min at 37°C. After the 30-min incubation, the medium used for infection was aspirated, and cells were washed once with PBS. Fresh DMEM containing 5% FBS was added to cells. At 13 h p.i., RFP-containing cells were counted using a Nexcelom Vision Image cytometer to determine the percentage of RFP-positive cells. The percentage of virus-infected cells was calculated by dividing the number of RFP-positive cells by the total number of cells counted.

### Effects of DEAE-dextran on VSV-p53wt (founder) and VSV-p53wt (SUIT-2) infectivity and replication.

BHK-21, SUIT-2, and MIA PaCa-2 cells were seeded into a 96-well plate to be approximately 100% confluent at the time of treatment and infection. Cells were washed once with PBS containing Mg^2+^ and Ca^2+^. Prior to infection, cells were pretreated with MEM without FBS (mock) or with 10 μg/ml DEAE-dextran in MEM without FBS for 30 min. After the 30-min pretreatment, VSV-p53wt (Founder) or VSV-p53wt (SUIT-2) in MEM with 0% FBS was directly added to cells at an MOI of either 1, 0.1, or 0.01 (MOI calculated based on virus titration on BHK-21) for 1 h at 37°C. Virus plus DEAE-dextran-containing medium was removed after the 1-h infection period, and fresh DMEM containing 5% FBS was added to cells. Cells were maintained at 37°C, and the level of VSV-encoded RFP fluorescence was measured over the course of 68 h p.i. using a fluorescence multiwell plate reader at the wavelength 590/645 nm.

## References

[1] Lyles DS, Rupprecht CE 2007 Rhabdoviridae, p 1363–1408. *In* Knipe DM, Howley PM, Griffin DE, Lamb RA, Martin MA, Roizman B, Strauss SE (ed), Fields virology, 5th ed Lippincott Williams & Wilkins, Philadelphia, PA.

[2] Hastie E, Cataldi M, Marriott I, Grdzelishvili VZ 2013 Understanding and altering cell tropism of vesicular stomatitis virus. Virus Res 176:16–32. doi:10.1016/j.virusres.2013.06.003. 23796410 PMC3865924

[3] Finkelshtein D, Werman A, Novick D, Barak S, Rubinstein M 2013 LDL receptor and its family members serve as the cellular receptors for vesicular stomatitis virus. Proc Natl Acad Sci U S A 110:7306–7311. doi:10.1073/pnas.1214441110. 23589850 PMC3645523

[4] Ammayappan A, Peng KW, Russell SJ 2013 Characteristics of oncolytic vesicular stomatitis virus displaying tumor-targeting ligands. J Virol 87:13543–13555. doi:10.1128/JVI.02240-13. 24089573 PMC3838279

[5] Amirache F, Levy C, Costa C, Mangeot PE, Torbett BE, Wang CX, Negre D, Cosset FL, Verhoeyen E 2014 Mystery solved: VSV-G-LVs do not allow efficient gene transfer into unstimulated T cells, B cells, and HSCs because they lack the LDL receptor. Blood 123:1422–1424. doi:10.1182/blood-2013-11-540641. 24578496

[6] Nikolic J, Belot L, Raux H, Legrand P, Gaudin Y, Albertini AA 2018 Structural basis for the recognition of LDL-receptor family members by VSV glycoprotein. Nat Commun 9:1029. doi:10.1038/s41467-018-03432-4. 29531262 PMC5847621

[7] Schlegel R, Tralka TS, Willingham MC, Pastan I 1983 Inhibition of VSV binding and infectivity by phosphatidylserine: is phosphatidylserine a VSV-binding site? Cell 32:639–646. doi:10.1016/0092-8674(83)90483-x. 6297804

[8] Coil DA, Miller AD 2004 Phosphatidylserine is not the cell surface receptor for vesicular stomatitis virus. J Virol 78:10920–10926. doi:10.1128/JVI.78.20.10920-10926.2004. 15452212 PMC521854

[9] Carneiro FA, Lapido-Loureiro PA, Cordo SM, Stauffer F, Weissmüller G, Bianconi ML, Juliano MA, Juliano L, Bisch PM, Da Poian AT 2006 Probing the interaction between vesicular stomatitis virus and phosphatidylserine. Eur Biophys J 35:145–154. doi:10.1007/s00249-005-0012-z. 16184389

[10] Schloemer RH, Wagner RR 1975 Cellular adsorption function of the sialoglycoprotein of vesicular stomatitis virus and its neuraminic acid. J Virol 15:882–893. 163924 10.1128/jvi.15.4.882-893.1975 PMC354532

[11] Guibinga GH, Miyanohara A, Esko JD, Friedmann T 2002 Cell surface heparan sulfate is a receptor for attachment of envelope protein-free retrovirus-like particles and VSV-G pseudotyped MLV-derived retrovirus vectors to target cells. Mol Ther 5:538–546. doi:10.1006/mthe.2002.0578. 11991744

[12] Hastie E, Grdzelishvili VZ 2012 Vesicular stomatitis virus as a flexible platform for oncolytic virotherapy against cancer. J Gen Virol 93:2529–2545. doi:10.1099/vir.0.046672-0. 23052398 PMC4091291

[13] Simovic B, Walsh SR, Wan Y 2015 Mechanistic insights into the oncolytic activity of vesicular stomatitis virus in cancer immunotherapy. Oncolytic Virother 4:157–167. doi:10.2147/OV.S66079. 27512679 PMC4918393

[14] Felt SA, Grdzelishvili VZ 2017 Recent advances in vesicular stomatitis virus-based oncolytic virotherapy: a 5-year update. J Gen Virol 98:2895–2911. doi:10.1099/jgv.0.000980. 29143726 PMC5845697

[15] Stojdl DF, Lichty BD, Knowles S, Marius R, Atkins H, Sonenberg N, Bell JC 2000 Exploiting tumor-specific defects in the interferon pathway with a previously unknown oncolytic virus. Nat Med 6:821–825. doi:10.1038/77558. 10888934

[16] Stojdl DF, Lichty BD, tenOever BR, Paterson JM, Power AT, Knowles S, Marius R, Reynard J, Poliquin L, Atkins H, Brown EG, Durbin RK, Durbin JE, Hiscott J, Bell JC 2003 VSV strains with defects in their ability to shutdown innate immunity are potent systemic anti-cancer agents. Cancer Cell 4:263–275. doi:10.1016/S1535-6108(03)00241-1. 14585354

[17] Lichty BD, Power AT, Stojdl DF, Bell JC 2004 Vesicular stomatitis virus: re-inventing the bullet. Trends Mol Med 10:210–216. doi:10.1016/j.molmed.2004.03.003. 15121047

[18] Barber GN 2004 Vesicular stomatitis virus as an oncolytic vector. Viral Immunol 17:516–527. doi:10.1089/vim.2004.17.516. 15671748

[19] Balachandran S, Barber GN 2004 Defective translational control facilitates vesicular stomatitis virus oncolysis. Cancer Cell 5:51–65. doi:10.1016/S1535-6108(03)00330-1. 14749126

[20] Moussavi M, Fazli L, Tearle H, Guo Y, Cox M, Bell J, Ong C, Jia W, Rennie PS 2010 Oncolysis of prostate cancers induced by vesicular stomatitis virus in PTEN knockout mice. Cancer Res 70:1367–1376. doi:10.1158/0008-5472.CAN-09-2377. 20145134

[21] Zhang KX, Matsui Y, Hadaschik BA, Lee C, Jia W, Bell JC, Fazli L, So AI, Rennie PS 2010 Down-regulation of type I interferon receptor sensitizes bladder cancer cells to vesicular stomatitis virus-induced cell death. Int J Cancer 127:830–838. doi:10.1002/ijc.25088. 19957332

[22] Marozin S, De Toni EN, Rizzani A, Altomonte J, Junger A, Schneider G, Thasler WE, Kato N, Schmid RM, Ebert O 2010 Cell cycle progression or translation control is not essential for vesicular stomatitis virus oncolysis of hepatocellular carcinoma. PLoS One 5:e10988. doi:10.1371/journal.pone.0010988. 20539760 PMC2881869

[23] Marozin S, Altomonte J, Stadler F, Thasler WE, Schmid RM, Ebert O 2008 Inhibition of the IFN-beta response in hepatocellular carcinoma by alternative spliced isoform of IFN regulatory factor-3. Mol Ther 16:1789–1797. doi:10.1038/mt.2008.201. 18781139

[24] Wang BX, Rahbar R, Fish EN 2011 Interferon: current status and future prospects in cancer therapy. J Interferon Cytokine Res 31:545–552. doi:10.1089/jir.2010.0158. 21323567

[25] Clarke DK, Nasar F, Lee M, Johnson JE, Wright K, Calderon P, Guo M, Natuk R, Cooper D, Hendry RM, Udem SA 2007 Synergistic attenuation of vesicular stomatitis virus by combination of specific G gene truncations and N gene translocations. J Virol 81:2056–2064. doi:10.1128/JVI.01911-06. 17151112 PMC1797571

[26] Johnson JE, Nasar F, Coleman JW, Price RE, Javadian A, Draper K, Lee M, Reilly PA, Clarke DK, Hendry RM, Udem SA 2007 Neurovirulence properties of recombinant vesicular stomatitis virus vectors in non-human primates. Virology 360:36–49. doi:10.1016/j.virol.2006.10.026. 17098273 PMC1865117

[27] Black BL, Lyles DS 1992 Vesicular stomatitis virus matrix protein inhibits host cell-directed transcription of target genes in vivo. J Virol 66:4058–4064. 1318397 10.1128/jvi.66.7.4058-4064.1992 PMC241208

[28] Black BL, Rhodes RB, McKenzie M, Lyles DS 1993 The role of vesicular stomatitis virus matrix protein in inhibition of host-directed gene expression is genetically separable from its function in virus assembly. J Virol 67:4814–4821. 8392615 10.1128/jvi.67.8.4814-4821.1993 PMC237868

[29] Coulon P, Deutsch V, Lafay F, Martinet-Edelist C, Wyers F, Herman RC, Flamand A 1990 Genetic evidence for multiple functions of the matrix protein of vesicular stomatitis virus. J Gen Virol 71:991–996. doi:10.1099/0022-1317-71-4-991. 2157808

[30] von Kobbe C, van Deursen JM, Rodrigues JP, Sitterlin D, Bachi A, Wu X, Wilm M, Carmo-Fonseca M, Izaurralde E 2000 Vesicular stomatitis virus matrix protein inhibits host cell gene expression by targeting the nucleoporin Nup98. Mol Cell 6:1243–1252. doi:10.1016/S1097-2765(00)00120-9. 11106761

[31] Ebert O, Harbaran S, Shinozaki K, Woo SL 2005 Systemic therapy of experimental breast cancer metastases by mutant vesicular stomatitis virus in immune-competent mice. Cancer Gene Ther 12:350–358. doi:10.1038/sj.cgt.7700794. 15565179

[32] Brown CW, Stephenson KB, Hanson S, Kucharczyk M, Duncan R, Bell JC, Lichty BD 2009 The p14 FAST protein of reptilian reovirus increases vesicular stomatitis virus neuropathogenesis. J Virol 83:552–561. doi:10.1128/JVI.01921-08. 18971262 PMC2612406

[33] Wollmann G, Rogulin V, Simon I, Rose JK, van den Pol AN 2010 Some attenuated variants of vesicular stomatitis virus show enhanced oncolytic activity against human glioblastoma cells relative to normal brain cells. J Virol 84:1563–1573. doi:10.1128/JVI.02040-09. 19906910 PMC2812324

[34] Ahmed M, McKenzie MO, Puckett S, Hojnacki M, Poliquin L, Lyles DS 2003 Ability of the matrix protein of vesicular stomatitis virus to suppress beta interferon gene expression is genetically correlated with the inhibition of host RNA and protein synthesis. J Virol 77:4646–4657. doi:10.1128/jvi.77.8.4646-4657.2003. 12663771 PMC152115

[35] Trottier MD, Lyles DS, Reiss CS 2007 Peripheral, but not central nervous system, type I interferon expression in mice in response to intranasal vesicular stomatitis virus infection. J Neurovirol 13:433–445. doi:10.1080/13550280701460565. 17994428 PMC2632583

[36] Heiber JF, Barber GN 2011 Vesicular stomatitis virus expressing tumor suppressor p53 is a highly attenuated, potent oncolytic agent. J Virol 85:10440–10450. doi:10.1128/JVI.05408-11. 21813611 PMC3187518

[37] Hastie E, Cataldi M, Steuerwald N, Grdzelishvili VZ 2015 An unexpected inhibition of antiviral signaling by virus-encoded tumor suppressor p53 in pancreatic cancer cells. Virology 483:126–140. doi:10.1016/j.virol.2015.04.017. 25965802 PMC4516574

[38] Okal A, Mossalam M, Matissek KJ, Dixon AS, Moos PJ, Lim CS 2013 A chimeric p53 evades mutant p53 transdominant inhibition in cancer cells. Mol Pharm 10:3922–3933. doi:10.1021/mp400379c. 23964676 PMC12186856

[39] Russell SJ, Peng KW, Bell JC 2012 Oncolytic virotherapy. Nat Biotechnol 30:658–670. doi:10.1038/nbt.2287. 22781695 PMC3888062

[40] Murphy AM, Besmer DM, Moerdyk-Schauwecker M, Moestl N, Ornelles DA, Mukherjee P, Grdzelishvili VZ 2012 Vesicular stomatitis virus as an oncolytic agent against pancreatic ductal adenocarcinoma. J Virol 86:3073–3087. doi:10.1128/JVI.05640-11. 22238308 PMC3302313

[41] Moerdyk-Schauwecker M, Shah NR, Murphy AM, Hastie E, Mukherjee P, Grdzelishvili VZ 2013 Resistance of pancreatic cancer cells to oncolytic vesicular stomatitis virus: role of type I interferon signaling. Virology 436:221–234. doi:10.1016/j.virol.2012.11.014. 23246628 PMC3544977

[42] Cataldi M, Shah NR, Felt SA, Grdzelishvili VZ 2015 Breaking resistance of pancreatic cancer cells to an attenuated vesicular stomatitis virus through a novel activity of IKK inhibitor TPCA-1. Virology 485:340–354. doi:10.1016/j.virol.2015.08.003. 26331681 PMC4619123

[43] Hastie E, Cataldi M, Moerdyk-Schauwecker MJ, Felt SA, Steuerwald N, Grdzelishvili VZ 2016 Novel biomarkers of resistance of pancreatic cancer cells to oncolytic vesicular stomatitis virus. Oncotarget 7:61601–61618. doi:10.18632/oncotarget.11202. 27533247 PMC5308675

[44] Felt SA, Droby GN, Grdzelishvili VZ 2017 Ruxolitinib and polycation combination treatment overcomes multiple mechanisms of resistance of pancreatic cancer cells to oncolytic vesicular stomatitis virus. J Virol 91:e00461-17. doi:10.1128/JVI.00461-17. 28566376 PMC5533928

[45] Felt SA, Moerdyk-Schauwecker MJ, Grdzelishvili VZ 2015 Induction of apoptosis in pancreatic cancer cells by vesicular stomatitis virus. Virology 474:163–173. doi:10.1016/j.virol.2014.10.026. 25463614 PMC4259820

[46] Steinhauer DA, Domingo E, Holland JJ 1992 Lack of evidence for proofreading mechanisms associated with an RNA virus polymerase. Gene 122:281–288. doi:10.1016/0378-1119(92)90216-c. 1336756

[47] Wertz GW, Moudy R, Ball LA 2002 Adding genes to the RNA genome of vesicular stomatitis virus: positional effects on stability of expression. J Virol 76:7642–7650. doi:10.1128/jvi.76.15.7642-7650.2002. 12097578 PMC136382

[48] Kandoth C, McLellan MD, Vandin F, Ye K, Niu B, Lu C, Xie M, Zhang Q, McMichael JF, Wyczalkowski MA, Leiserson MD, Miller CA, Welch JS, Walter MJ, Wendl MC, Ley TJ, Wilson RK, Raphael BJ, Ding L 2013 Mutational landscape and significance across 12 major cancer types. Nature 502:333–339. doi:10.1038/nature12634. 24132290 PMC3927368

[49] Bressy C, Hastie E, Grdzelishvili VZ 2017 Combining oncolytic virotherapy with p53 tumor suppressor gene therapy. Mol Ther Oncolytics 5:20–40. doi:10.1016/j.omto.2017.03.002. 28480326 PMC5415316

[50] Kuhn I, Harden P, Bauzon M, Chartier C, Nye J, Thorne S, Reid T, Ni S, Lieber A, Fisher K, Seymour L, Rubanyi GM, Harkins RN, Hermiston TW 2008 Directed evolution generates a novel oncolytic virus for the treatment of colon cancer. PLoS One 3:e2409. doi:10.1371/journal.pone.0002409. 18560559 PMC2423470

[51] Yan W, Kitzes G, Dormishian F, Hawkins L, Sampson-Johannes A, Watanabe J, Holt J, Lee V, Dubensky T, Fattaey A, Hermiston T, Balmain A, Shen Y 2003 Developing novel oncolytic adenoviruses through bioselection. J Virol 77:2640–2650. doi:10.1128/jvi.77.4.2640-2650.2003. 12552003 PMC141112

[52] Kuhn I, Bauzon M, Green N, Seymour L, Fisher K, Hermiston T 2017 OvAd1, a novel, potent, and selective chimeric oncolytic virus developed for ovarian cancer by 3D-directed evolution. Mol Ther Oncolytics 4:55–66. doi:10.1016/j.omto.2016.12.001. 28345024 PMC5363728

[53] Svyatchenko VA, Ternovoy VA, Kiselev NN, Demina AV, Loktev VB, Netesov SV, Chumakov PM 2017 Bioselection of coxsackievirus B6 strain variants with altered tropism to human cancer cell lines. Arch Virol 162:3355–3362. doi:10.1007/s00705-017-3492-0. 28766058

[54] Sanjuan R, Grdzelishvili VZ 2015 Evolution of oncolytic viruses. Curr Opin Virol 13:1–5. doi:10.1016/j.coviro.2015.01.014. 25699475

[55] Zainutdinov SS, Kochneva GV, Netesov SV, Chumakov PM, Matveeva OV 2019 Directed evolution as a tool for the selection of oncolytic RNA viruses with desired phenotypes. Oncolytic Virother 8:9–26. doi:10.2147/OV.S176523. 31372363 PMC6636189

[56] Bauzon M, Hermiston TW 2012 Oncolytic viruses: the power of directed evolution. Adv Virol 2012:586389. doi:10.1155/2012/586389. 22312363 PMC3265225

[57] Novella IS 2003 Contributions of vesicular stomatitis virus to the understanding of RNA virus evolution. Curr Opin Microbiol 6:399–405. doi:10.1016/S1369-5274(03)00084-5. 12941412

[58] Hernandez-Alonso P, Garijo R, Cuevas JM, Sanjuan R 2015 Experimental evolution of an RNA virus in cells with innate immunity defects. Virus Evol 1:vev008. doi:10.1093/ve/vev008. 27774280 PMC5014476

[59] Yu JH, Schaffer DV 2006 Selection of novel vesicular stomatitis virus glycoprotein variants from a peptide insertion library for enhanced purification of retroviral and lentiviral vectors. J Virol 80:3285–3292. doi:10.1128/JVI.80.7.3285-3292.2006. 16537595 PMC1440395

[60] Davis JN, van den Pol AN 2010 Viral mutagenesis as a means for generating novel proteins. J Virol 84:1625–1630. doi:10.1128/JVI.01747-09. 19906913 PMC2812339

[61] Garijo R, Hernández-Alonso P, Rivas C, Diallo J-S, Sanjuán R 2014 Experimental evolution of an oncolytic vesicular stomatitis virus with increased selectivity for p53-deficient cells. PLoS One 9:e102365. doi:10.1371/journal.pone.0102365. 25010337 PMC4092128

[62] Gao Y, Whitaker-Dowling P, Watkins SC, Griffin JA, Bergman I 2006 Rapid adaptation of a recombinant vesicular stomatitis virus to a targeted cell line. J Virol 80:8603–8612. doi:10.1128/JVI.00142-06. 16912309 PMC1563842

[63] Janelle V, Brassard F, Lapierre P, Lamarre A, Poliquin L 2011 Mutations in the glycoprotein of vesicular stomatitis virus affect cytopathogenicity: potential for oncolytic virotherapy. J Virol 85:6513–6520. doi:10.1128/JVI.02484-10. 21561919 PMC3126483

[64] Wollmann G, Tattersall P, van den Pol AN 2005 Targeting human glioblastoma cells: comparison of nine viruses with oncolytic potential. J Virol 79:6005–6022. doi:10.1128/JVI.79.10.6005-6022.2005. 15857987 PMC1091699

[65] Shcherbo D, Merzlyak EM, Chepurnykh TV, Fradkov AF, Ermakova GV, Solovieva EA, Lukyanov KA, Bogdanova EA, Zaraisky AG, Lukyanov S, Chudakov DM 2007 Bright far-red fluorescent protein for whole-body imaging. Nat Methods 4:741–746. doi:10.1038/nmeth1083. 17721542

[66] Bressy C, Droby GN, Maldonado BD, Steuerwald N, Grdzelishvili VZ 2018 Cell cycle arrest in G_2_/M phase enhances replication of interferon-sensitive cytoplasmic RNA viruses via inhibition of antiviral gene expression. J Virol 93:e01885-18. doi:10.1128/JVI.01885-18. PMC6364032 30487274

[67] Otsuki K, Maeda J, Yamamoto H, Tsubokura M 1979 Studies on avian infectious bronchitis virus (IBV). III. Interferon induction by and sensitivity to interferon of IBV. Arch Virol 60:249–255. doi:10.1007/bf01317496. 228636 PMC7087316

[68] Habjan M, Penski N, Spiegel M, Weber F 2008 T7 RNA polymerase-dependent and -independent systems for cDNA-based rescue of Rift Valley fever virus. J Gen Virol 89:2157–2166. doi:10.1099/vir.0.2008/002097-0. 18753225

[69] Furukawa T, Duguid WP, Rosenberg L, Viallet J, Galloway DA, Tsao MS 1996 Long-term culture and immortalization of epithelial cells from normal adult human pancreatic ducts transfected by the E6E7 gene of human papilloma virus 16. Am J Pathol 148:1763–1770. 8669463 PMC1861644

[70] Boritz E, Gerlach J, Johnson JE, Rose JK 1999 Replication-competent rhabdoviruses with human immunodeficiency virus type 1 coats and green fluorescent protein: entry by a pH-independent pathway. J Virol 73:6937–6945. 10400792 10.1128/jvi.73.8.6937-6945.1999 PMC112779

[71] Davis HE, Rosinski M, Morgan JR, Yarmush ML 2004 Charged polymers modulate retrovirus transduction via membrane charge neutralization and virus aggregation. Biophys J 86:1234–1242. doi:10.1016/S0006-3495(04)74197-1. 14747357 PMC1303915

[72] Pizzato M, Marlow SA, Blair ED, Takeuchi Y 1999 Initial binding of murine leukemia virus particles to cells does not require specific Env-receptor interaction. J Virol 73:8599–8611. 10482613 10.1128/jvi.73.10.8599-8611.1999 PMC112880

[73] Sharma S, Miyanohara A, Friedmann T 2000 Separable mechanisms of attachment and cell uptake during retrovirus infection. J Virol 74:10790–10795. doi:10.1128/jvi.74.22.10790-10795.2000. 11044124 PMC110954

[74] Reiser J, Harmison G, Kluepfel-Stahl S, Brady RO, Karlsson S, Schubert M 1996 Transduction of nondividing cells using pseudotyped defective high-titer HIV type 1 particles. Proc Natl Acad Sci U S A 93:15266–15271. doi:10.1073/pnas.93.26.15266. 8986799 PMC26392

[75] Yee JK, Friedmann T, Burns JC 1994 Generation of high-titer pseudotyped retroviral vectors with very broad host range. Methods Cell Biol 43:99–112. doi:10.1016/S0091-679X(08)60600-7. 7823872

[76] Denning W, Das S, Guo S, Xu J, Kappes JC, Hel Z 2013 Optimization of the transductional efficiency of lentiviral vectors: effect of sera and polycations. Mol Biotechnol 53:308–314. doi:10.1007/s12033-012-9528-5. 22407723 PMC3456965

[77] Roche S, Bressanelli S, Rey FA, Gaudin Y 2006 Crystal structure of the low-pH form of the vesicular stomatitis virus glycoprotein G. Science 313:187–191. doi:10.1126/science.1127683. 16840692

[78] Roche S, Rey FA, Gaudin Y, Bressanelli S 2007 Structure of the prefusion form of the vesicular stomatitis virus glycoprotein G. Science 315:843–848. doi:10.1126/science.1135710. 17289996

[79] Akkina RK, Walton RM, Chen ML, Li QX, Planelles V, Chen IS 1996 High-efficiency gene transfer into CD34^+^ cells with a human immunodeficiency virus type 1-based retroviral vector pseudotyped with vesicular stomatitis virus envelope glycoprotein G. J Virol 70:2581–2585. 8642689 10.1128/jvi.70.4.2581-2585.1996 PMC190105

[80] Burns JC, Friedmann T, Driever W, Burrascano M, Yee JK 1993 Vesicular stomatitis virus G glycoprotein pseudotyped retroviral vectors: concentration to very high titer and efficient gene transfer into mammalian and nonmammalian cells. Proc Natl Acad Sci U S A 90:8033–8037. doi:10.1073/pnas.90.17.8033. 8396259 PMC47282

[81] Matlin KS, Reggio H, Helenius A, Simons K 1982 Pathway of vesicular stomatitis virus entry leading to infection. J Mol Biol 156:609–631. doi:10.1016/0022-2836(82)90269-8. 6288961

[82] Conti C, Mastromarino P, Riccioli A, Orsi N 1991 Electrostatic interactions in the early events of VSV infection. Res Virol 142:17–24. doi:10.1016/0923-2516(91)90023-v. 1647050

[83] Bailey CA, Miller DK, Lenard J 1984 Effects of DEAE-dextran on infection and hemolysis by VSV. Evidence that nonspecific electrostatic interactions mediate effective binding of VSV to cells. Virology 133:111–118. doi:10.1016/0042-6822(84)90429-x. 6199890

[84] Davis HE, Morgan JR, Yarmush ML 2002 Polybrene increases retrovirus gene transfer efficiency by enhancing receptor-independent virus adsorption on target cell membranes. Biophys Chem 97:159–172. doi:10.1016/s0301-4622(02)00057-1. 12050007

[85] Rose JK, Gallione CJ 1981 Nucleotide sequences of the mRNA’s encoding the vesicular stomatitis virus G and M proteins determined from cDNA clones containing the complete coding regions. J Virol 39:519–528. 6268840 10.1128/jvi.39.2.519-528.1981 PMC171362

[86] Robertson MA, Etchison JR, Robertson JS, Summers DF, Stanley P 1978 Specific changes in the oligosaccharide moieties of VSV grown in different lectin-resistant CHO cells. Cell 13:515–526. doi:10.1016/0092-8674(78)90325-2. 207434

[87] Etchison JR, Holland JJ 1974 Carbohydrate composition of the membrane glycoprotein of vesicular stomatitis virus grown in four mammalian cell lines. Proc Natl Acad Sci U S A 71:4011–4014. doi:10.1073/pnas.71.10.4011. 4372602 PMC434317

[88] Reading CL, Penhoet EE, Ballou CE 1978 Carbohydrate structure of vesicular stomatitis virus glycoprotein. J Biol Chem 253:5600–5612. 209045

[89] Ortega V, Stone JA, Contreras EM, Iorio RM, Aguilar HC 2019 Addicted to sugar: roles of glycans in the order Mononegavirales. Glycobiology 29:2–21. doi:10.1093/glycob/cwy053. 29878112 PMC6291800

[90] Farley DC, Iqball S, Smith JC, Miskin JE, Kingsman SM, Mitrophanous KA 2007 Factors that influence VSV-G pseudotyping and transduction efficiency of lentiviral vectors—in vitro and in vivo implications. J Gene Med 9:345–356. doi:10.1002/jgm.1022. 17366519

[91] Puri A, Grimaldi S, Blumenthal R 1992 Role of viral envelope sialic acid in membrane fusion mediated by the vesicular stomatitis virus envelope glycoprotein. Biochemistry 31:10108–10113. doi:10.1021/bi00156a034. 1327132

[92] Stanley P, Vivona G, Atkinson PH 1984 1H NMR spectroscopy of carbohydrates from the G glycoprotein of vesicular stomatitis virus grown in parental and Lec4 Chinese hamster ovary cells. Arch Biochem Biophys 230:363–374. doi:10.1016/0003-9861(84)90119-X. 6324683

[93] Marozin S, Altomonte J, Apfel S, Dinh PX, De Toni EN, Rizzani A, Nussler A, Kato N, Schmid RM, Pattnaik AK, Ebert O 2012 Posttranslational modification of vesicular stomatitis virus glycoprotein, but not JNK inhibition, is the antiviral mechanism of SP600125. J Virol 86:4844–4855. doi:10.1128/JVI.06649-11. 22345438 PMC3347359

[94] Lauring AS, Frydman J, Andino R 2013 The role of mutational robustness in RNA virus evolution. Nat Rev Microbiol 11:327–336. doi:10.1038/nrmicro3003. 23524517 PMC3981611

[95] Quinones-Kochs MI, Schnell MJ, Buonocore L, Rose JK 2001 Mechanisms of loss of foreign gene expression in recombinant vesicular stomatitis viruses. Virology 287:427–435. doi:10.1006/viro.2001.1058. 11531419

[96] Collins PL, Bukreyev A, Murphy BR 2008 What are the risks—hypothetical and observed—of recombination involving live vaccines and vaccine vectors based on nonsegmented negative-strain RNA viruses? J Virol 82:9805–9806. doi:10.1128/JVI.01336-08. 18796655 PMC2546970

[97] Pfaller CK, Cattaneo R, Schnell MJ 2015 Reverse genetics of Mononegavirales: how they work, new vaccines, and new cancer therapeutics. Virology 479–480:331–344. doi:10.1016/j.virol.2015.01.029. PMC4557643 25702088

[98] Schnell MJ, Buonocore L, Kretzschmar E, Johnson E, Rose JK 1996 Foreign glycoproteins expressed from recombinant vesicular stomatitis viruses are incorporated efficiently into virus particles. Proc Natl Acad Sci U S A 93:11359–11365. doi:10.1073/pnas.93.21.11359. 8876140 PMC38062

[99] Dinh PX, Panda D, Das PB, Das SC, Das A, Pattnaik AK 2012 A single amino acid change resulting in loss of fluorescence of eGFP in a viral fusion protein confers fitness and growth advantage to the recombinant vesicular stomatitis virus. Virology 432:460–469. doi:10.1016/j.virol.2012.07.004. 22832124 PMC3423531

[100] Roberts A, Reuter JD, Wilson JH, Baldwin S, Rose JK 2004 Complete protection from papillomavirus challenge after a single vaccination with a vesicular stomatitis virus vector expressing high levels of L1 protein. J Virol 78:3196–3199. doi:10.1128/jvi.78.6.3196-3199.2004. 14990742 PMC353748

[101] van den Pol AN, Davis JN 2013 Highly attenuated recombinant vesicular stomatitis virus VSV-12’GFP displays immunogenic and oncolytic activity. J Virol 87:1019–1034. doi:10.1128/JVI.01106-12. 23135719 PMC3554062

[102] Mebatsion T, Schnell MJ, Cox JH, Finke S, Conzelmann KK 1996 Highly stable expression of a foreign gene from rabies virus vectors. Proc Natl Acad Sci U S A 93:7310–7314. doi:10.1073/pnas.93.14.7310. 8692989 PMC38980

[103] Hudacek AW, Al-Saleem FH, Willet M, Eisemann T, Mattis JA, Simpson LL, Schnell MJ 2014 Recombinant rabies virus particles presenting botulinum neurotoxin antigens elicit a protective humoral response in vivo. Mol Ther Methods Clin Dev 1:14046. doi:10.1038/mtm.2014.46. 26015984 PMC4362357

[104] Schnell MJ, Buonocore L, Whitt MA, Rose JK 1996 The minimal conserved transcription stop-start signal promotes stable expression of a foreign gene in vesicular stomatitis virus. J Virol 70:2318–2323. 8642658 10.1128/jvi.70.4.2318-2323.1996 PMC190073

[105] Moerdyk-Schauwecker M, Hwang SI, Grdzelishvili VZ 2014 Cellular proteins associated with the interior and exterior of vesicular stomatitis virus virions. PLoS One 9:e104688. doi:10.1371/journal.pone.0104688. 25105980 PMC4126742

[106] Iwamura T, Katsuki T, Ide K 1987 Establishment and characterization of a human pancreatic cancer cell line (SUIT-2) producing carcinoembryonic antigen and carbohydrate antigen 19-9. Jpn J Cancer Res 78:54–62. 3102439

